# A universal strategy for the fabrication of single-photon and multiphoton NIR nanoparticles by loading organic dyes into water-soluble polymer nanosponges

**DOI:** 10.1186/s12951-022-01515-5

**Published:** 2022-07-06

**Authors:** Li-Xing Yang, Yu-Cheng Liu, Chang-Hui Cho, Yi-Rou Chen, Chan-Shan Yang, Yin-Lin Lu, Zhiming Zhang, Yi-Tseng Tsai, Yu-Cheng Chin, Jiashing Yu, Hsiu-Min Pan, Wei-Rou Jiang, Zi-Chun Chia, Wei-Shiang Huang, Yu-Lin Chiu, Chun-Kai Sun, Yu-Ting Huang, Li-Ming Chen, Ken-Tsung Wong, Han-Min Huang, Chih-Hsin Chen, Yuan Jay Chang, Chih-Chia Huang, Tzu-Ming Liu

**Affiliations:** 1 Department of Photonics, National Cheng Kung University, Tainan, 70101 Taiwan; 2 Institute of Translational Medicine, Faculty of Health Sciences & Ministry of Education Frontiers Science Center for Precision Oncology, University of Macau, Macao SAR, 999078 China; 3 Department of Chemistry, Tunghai University, Taichung, 40704 Taiwan; 4 Department of Chemistry, Tamkang University, 25137, New Taipei City, Taiwan; 5 Institute and Undergraduate Program of Electro-Optical Engineering, National Taiwan Normal University, Taipei, 11677 Taiwan; 6 Department of Chemical Engineering, National Taiwan University, Taipei, 106 Taiwan; 7 Department of Chemistry, National Taiwan University, Taipei, 10617 Taiwan; 8 Center of Applied Nanomedicine, National Cheng Kung University, Tainan, 70101 Taiwan

**Keywords:** Polymer, Near-infrared red, Optoelectronic material, Photodynamic therapy, Theranostic, Multiphoton microscopy, Fluorescence imaging

## Abstract

The development of optical organic nanoparticles (NPs) is desirable and widely studied. However, most organic dyes are water-insoluble such that the derivatization and modification of these dyes are difficult. Herein, we demonstrated a simple platform for the fabrication of organic NPs designed with emissive properties by loading ten different organic dyes (molar masses of 479.1–1081.7 g/mol) into water-soluble polymer nanosponges composed of poly(styrene-alt-maleic acid) (PSMA). The result showed a substantial improvement over the loading of commercial dyes (3.7–50% loading) while preventing their spontaneous aggregation in aqueous solutions. This packaging strategy includes our newly synthesized organic dyes (> 85% loading) designed for OPVs (242), DSSCs (YI-1, YI-3, YI-8), and OLEDs (ADF-1–3, and DTDPTID) applications. These low-cytotoxicity organic NPs exhibited tunable fluorescence from visible to near-infrared (NIR) emission for cellular imaging and biological tracking in vivo. Moreover, PSMA NPs loaded with designed NIR-dyes were fabricated, and photodynamic therapy with these dye-loaded PSMA NPs for the photolysis of cancer cells was achieved when coupled with 808 nm laser excitation. Indeed, our work demonstrates a facile approach for increasing the biocompatibility and stability of organic dyes by loading them into water-soluble polymer-based carriers, providing a new perspective of organic optoelectronic materials in biomedical theranostic applications.

**Supplementary Information:**

The online version contains supplementary material available at 10.1186/s12951-022-01515-5.

## Introduction

Over the past several decades, numerous organic molecules with near-infrared (NIR) optical properties have been synthesized for the development of efficient optoelectronic devices, such as organic light-emitting diodes (OLEDs) [1, 2], organic photovoltaics (OPVs) [3, 4], and dye-sensitized solar cells (DSSCs) [5, 6]. Organic NIR dyes applied in organic electronic devices are suitable for biomedical applications due to their distinct optical property and excellent photostability. Rare earth (lanthanide) complexes [7], transition metal complexes [8], as well as organic molecules with large dipole moment [9] have been found to exhibit extremely low-bandgap and NIR emission [10–12]. The studies of organic dyes as NIR emitters have gained attention recently due to their practical biomedical applications usage. However, NIR organic dyes are significantly limited by their low solubility in the aqueous phase and low biocompatibility in biological environments.

The fabrication of NIR nanoparticles (NPs) is of great significance in biological researches because they could provide more detailed information at the molecular level such as bio-imaging, cancer therapy, and image-guided drug delivery [13–15]. In addition, hydrophilic NIR NPs possess higher biocompatibility and water solubility than free NIR dyes, such that they are suitable for biomedical applications. Unfortunately, the extended π-conjugated system from free NIR dyes usually results in a bulky molecular structure which lowers the efficiency of the NPs fabrication using these molecules [13–23]. In addition, the synthetic conditions for fabricating NIR NPs from NIR dyes are difficult to control, especially when -COOH or -NH_2_ functional groups are needed for subsequent biografting/derivatization without changing their optical properties after bioconjugation [24–27]. Based on the above challenges, ideal methods for fabricating NIR NPs are critically important for the NIR dyes in practical applications.

In recent studies, several methods for the fabrication of NIR NPs have been developed and investigated [15, 18, 21–23, 28–34]. These methods include emulsion-solvent evaporation [20], polymer grafting [21, 29, 30, 32], AIE self-assembling [23, 31], and coprecipitation [18, 22]. On the other hand, scientifically formulated conjugation strategies by incorporating π-conjugated backbones as the main chain or NIR dyes on the side chain have led to the synthesis of NIR semiconducting polymers in the nanoscale [33, 34]. Unfortunately, due to the structural diversity of different organic NIR dyes, the structural modification strategy for each NIR dye for the fabrication of NPs must be tailor-made. Therefore, the synthetic procedures for these NPs are complicated and may be subject to batch-to-batch variation during mass production [2, 35]. These obstacles impede the widespread use of organic dyes as NPs in biological experiments. For the future exploration of organic dyes in biomedical applications, developing a packaging strategy is simple and facile method for the fabrication of dye-based NPs that is compatible with different dye molecular structures is highly desirable.

**Fig. 1:**
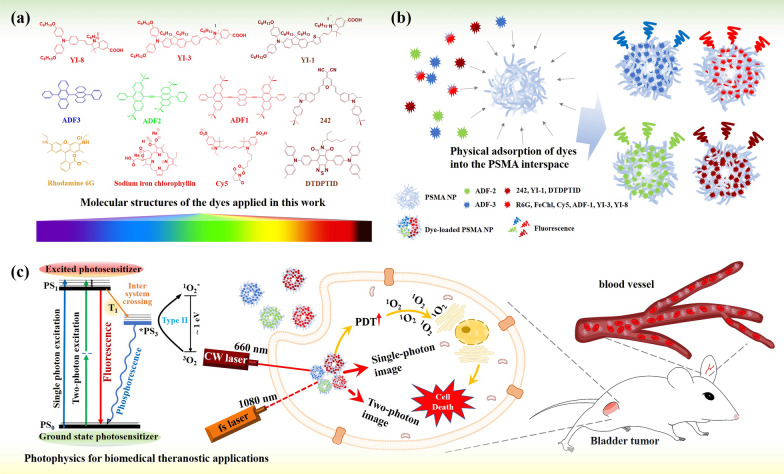
**a** Chemical structures and their corresponding fluorescence regions of organic dyes, including OPVs [242 (NIR)], DSSCs [YI-1 (NIR), YI-3 (red), YI-8 (red)], OLEDs [ADF1 (red), ADF-2 (green), ADF-3 (blue), and DTDPTID (NIR)], and commercial materials [Rhodamine 6G (R6G) (orange-red), sodium iron chlorophyllin (FeChl) (red), and CY5 (red)]. **b** Schematic illustration for the sponge-like swelling nature of the PSMA NPs capable of physical adsorption these photosensitive molecules into the interspace. **c** The utility of the CW and fs laser to make the dye-loaded PSMA NPs fluorescent induction (via single-photon and two-photon absorption) of single-photon/multiphoton imaging in cellular and blood vessel and NIR-PDT (via an intersystem crossing pathway) in bladder tumor

In this work, we developed a universal strategy to fabricate optically active NPs by loading organic dyes into metal-free poly(styrene-alt-maleic acid) (PSMA) NPs (Fig. 1a, b). The PSMA organic NPs exhibit sponge properties for the direct adsorption of commercially available dyes with 3.7–50% of dye loading, and in our newly synthesized organic dyes designed for OPVs (242), DSSCs (YI-1, YI-3, YI-8), and OLEDs (ADF-1–3, DTDPTID) applications showed dye loading efficiency over 85%. We demonstrated that this strategy provides design flexibility for loading the dyes with the visible to NIR region emission. These PSMA-based nanosponges showed physisorption ability, which prevented the aggregation of the organic molecules in water and enhanced the biocompatibility [19] and conjugation/complex capability [17, 36] due to their amphiphilic nature in aqueous solutions [16, 17, 36–39]. Our technique not only satisfies the need for the self-assembly of dyes to facilitate the formation of a micelle configuration, which has been shown to be susceptible to environmental dissociation [40], but it is also not limited to the adsorbing positively charged dyes [36, 41]. Owing to the single-photon and multiphoton NIR fluorescence properties of the organic dyes, NIR bioimaging in vitro and in vivo using these organic NPs was achieved (Fig. 1c). In addition, the NIR-triggered photodynamic therapy (PDT) of bladder cancer cells was demonstrated by using PSMA NPs loaded with NIR dye.

## Materials and methods

### Materials

Poly(styrene-alt-maleic acid) sodium salt solution (PSMA; Sigma–Aldrich), CuCl_2_ (J.T. Baker), N_2_H_4_ (Alfa Aesar), hydrochloric acid (HCl, 37%; Fluka), H_2_N-PEG-NH_2_ (MW = 3500; JenKem Technology), 1-ethyl-3-(-3-dimethylamiopropyl)carbodiimide hydrochloride (EDC; Matrix scientific), N-hydroxysuccinimide (NHS, 98%+ ; Alfa Aesar), 4-carboxyphenylboronic acid (CPBA; Aldrich), 3-(4,5-dimethylthiazol-2-yl)-2,5-diphenyltetrazolium bromide (MTT assay reagent; Alfa Aesar), 2,7-dichlorofluorescin diacetate (DCFH-DA; Sigma–Aldrich), N,N-dimethyl-4-nitrosoaniline (RNO; Alfa Aesar), imidazole (Acros organics), dimethyl sulfoxide (DMSO; Fisher scientific), sodium iron chlorophyllin salt (FeChl), rhodamine 6G (R6G; Sigma–Aldrich), cyanine 5 (Cy5; Tocris Bioscience), tetrahydrofuran (THF; Macron Fine Chemicals), ethanol (J.T. Baker), and 5,5′,6,6′-tetrachloro-1,1′,3,3′-tetraethylbenzimidazolylcarbocyanine (JC-1; Sigma–Aldrich). The molecular structures of the OLED-(ADF-1–3 and DTDPTID), OPV-(242), and DSSC-(YI-1, YI-3, YI-8) based photosensitive molecules were shown in Fig. 1a. 4,8-Dibromo-6-(2-ethylhexyl)-5*H*-[1,2,5]thiadiazolo[3,4-*f*]isoindole-5,7(*6H*)-dione (TID-Br) was prepared according to the literature [42]. For the synthesis of DTDPTID, please see the SI experiment section. These organic chromophore structures were first published in this work, and the detailed synthesis methods in the SI experiment section.

### Characterization

Transmission electron microscopy (TEM, Hitachi H7500 TEM instrument at 80 kV) was utilized to determine the structures and sizes of the PSMA-dye NPs. To prepare the negative stain TEM imaging, the carbon support grids were first processed with plasma to make the surface hydrophilic to absorb the sample. The prepared PSMA-dye samples were dropped onto the grid for 1 min to absorb the NPs. The excess solution was removed, followed by staining with 2% uranyl acetate for 1 min to precipitate the uranium around the NPs for contrast enhancement. The prepared negative stained samples were measured by TEM (JEM-1400, JEOL, Japan) for accessing the PSMA particle structure before and after the dye loading. The absorption spectra of the PSMA-dye NP samples were measured by a V-730 UV–Vis spectrophotometer from Jasco (USA). The absorption rates of the PSMA dye samples were quantified by a fluorescence microplate reader (Biotek Synergy H1, USA). The particle sizes by dynamic light scattering (DLS) and zeta potentials (HORIBA, Ltd., Japan) of the PSMA-dye NP samples dispersed in an aqueous solution were measured. The FT-IR spectra obtained by Fourier transform infrared spectroscopy (JASCO FT/IR-4700) with a KBr plate were used for the vibrational signal analyses of the PSMA-dye NP samples. A fluorescence microscope (Olympus IX73) was employed for fluorescent cell imaging with UFUNA (420–460 nm), UFBWA (510–550 nm), and UFGW (> 575 nm) filters for blue, green, and red fluorescence observation, respectively. A Nikon Inverted Multiphoton Microscope Eclipse (A1MP + Eclipse Ti-2E, Nikon instrument Inc., Japan) with a 40 × NA = 1.15 water-immersion objective was used for in vitro and in vivo nonlinear photonics analysis.

### PSMA NP preparation

The PSMA NPs were prepared through the etching of Cu@PSMA NPs by HCl solution. In brief, 1 mL of CuCl_2_ solution (6 mM) was first mixed with 10 mL of PSMA solution (6 mg/mL) and then 22 μL of HCl (2 M) and 0.1 mL of N_2_H_4_ hydrate were subsequently added to the mixture before being sealed in a 23 mL Teflon-lined autoclave reactor. The sealed reactor was subjected to hydrothermal treatment at 158 °C for 3 h in an oven to form Cu@PSMA NPs. The Cu@PSMA NPs were etched by 1.5 M HCl for 1.5 h to resolve the copper, resulting in PSMA NPs. Finally, the resulting product was collected by centrifuging at 11,000 rpm for 10 min to collect the PSMA NPs, followed by three centrifugation/resuspension processes for product purification.

### PSMA-dye NP preparation

Small molecular dyes (FeChl, Cy5 and R6G) and OLED-, OPV-, and DSSC-based photosensitive molecules were prepared in THF with 5 mM stock solutions. The prepared PSMA NPs (OD = 2 at 230 nm) were mixed with 0.2 mM of each dye in a 1:1 volume ratio and kept in the dark at 4 °C overnight for dye adsorption. The synthesized PSMA-dye NPs were then centrifuged at 11,000 rpm for 10 min to remove the nonadsorbed dyes and washed with water three times. The absorption rates of the immobilized dyes were determined by using a fluorescent microplate reader.

### CPBA-conjugated PSMA-dye NP preparation

To enhance the in vitro PDT of bladder cancer cells, we prepared PSMA-dye NPs conjugated with the target ligand CPBA. Briefly, 4 mg of NH_2_-PEG-NH_2_ was mixed with 200 µL of PSMA-dye NPs (0.1 mM of dyes and ultrasonicated in the dark for 5 min. Then, 0.7 mL of NHS/EDC mixture (1.5 mg/1.5 mg) was added to induce PEGylation, followed by mixing with CPBA (0.05 mM, 50 µL) in the dark at 4 °C for 1 h. The sample was then centrifuged at 9500 rpm for 10 min and washed with DI water three times to obtain CPBA-modified PSMA-dye NPs for the in vitro PDT test.

### Singlet oxygen detection

Different dyes and dye-immobilized PSMA NPs (0.1 mM) were thoroughly mixed with RNO (0.05 mM) and imidazole (0.05 mM) and then exposed under a 660 nm LED board with a 75 mW/cm^2^ power density or an 808 nm continuous-wave (CW) laser with a power density of 0.9 W/cm^2^ for 10 min. For singlet oxygen detection, the decay of the RNO absorption peak at 440 nm was measured every 2 min during the exposure time.

### Temperature examination under 808 nm laser irradiation

The temperature elevation of the dye-immobilized PSMA NPs was measured by placing the material solutions (0.1 mM) in 96-well plates and irradiating them with an 808 nm CW laser with a power density of 0.9 W/cm^2^. A thermocouple was immersed in the material solutions to monitor the temperature and record the thermal curve.

### Cell culture and cell viability assays

MB49 mouse bladder cancer cells were cultured in Roswell Park Memorial Institute (RPMI) medium 1640 supplemented with 10% fetal bovine serum (FBS) and 1% antibiotics in a humidified incubator at 37 °C with air containing 5% CO_2_. MTT assays were performed to estimate cell viability after the cells were treated with different PSMA-dye NPs without light irradiation. All experiments were performed with 8 × 10^3^ cells/well in a 96-well culture plate overnight. Then, the cells were treated with different types and concentrations of PSMA-dye NPs and incubated for 24 h. The medium was then replaced with fresh medium containing MTT reagent (0.5 mg/mL), and the cells were incubated at 37 °C for another 1 h. The medium was then removed, and the converted formazan was dissolved in DMSO and quantified by optical absorbance at 565 nm to determine the cell viability.

### Mitochondria membrane potential loss analysis

The membrane potential status of mitochondria was detected using JC-1 reagent. The aggregate form of JC-1 in normal mitochondria has absorption and emission maxima at 585 and 590 nm, respectively, while the maxima for its monomer form in damaged mitochondria occur at 510 and 527 nm. A stock solution of JC-1 was prepared by dissolving 5 mg of JC-1 in 1 mL of DMSO followed by storage at − 20 °C. Cells were seeded at a density of 8 × 10^4^ cells in 3.5 cm dishes without PSMA-dye NPs and coincubated in JC-1-containing medium (5 µg/mL) for 30 min at 37 °C. After washing three times with culture medium, the cells were observed and analyzed by fluorescence microscopy (Olympus IX73).

### Characterization of multiphoton fluorescence spectra and two-photon absorption coefficient for PSMA-242, PSMA-YI-1, PSMA-ADF1, and PSMA-DTDPTID

To evaluate the multiphoton fluorescence emission features of NPs prepared with different NP formulations (PSMA-242, 72 ppm; PSMA-YI-1, 118 ppm; PSMA-ADF1, 82 ppm; PSMA-DTDPTID, 217 ppm), these NPs were characterized by a CCD-cooled spectrometer (iDus 401 plus shamrock 193i, ANDOR, Oxford Instruments) under laser radiation at different wavelengths. To measure the two-photon absorption coefficient of the chromophore center, we employed the open aperture Z-scan technique. The sample cell was constructed by sandwiching the NPs and PSMA NPs between two optical-grade fused silica windows. The thickness of NPs layer was controlled with a 600 µm Mylar spacer. A general Z-scan system as [43] was adopted by applying a Ti: sapphire laser (Coherent Legend Elite HE+) with pulse width and repetition rate of 35 fs and 5 kHz, respectively. By translating the sample across the focal point, we could measure the intensity dependent absorption data. With a model fitting, we could obtain the two-photon absorption (TPA) coefficient *β*. On the other hand, the relation between TPA cross section, *σ* (cm^4^ s/photon/molecule) and *β* is *σ* = *ħωβ*/ (*N*_A_*ρ* × 10^–3^) [43], where *ħ*, *ω*, N_A_, and *ρ* is the reduced Plank constant, the angular frequency, Avogadro’s number, and the concentration in mole per liter, respectively. In other words, the *σ* is directly positively proportional to the *β*.

### Multiphoton cellular imaging

MDA-MB-231 cells (2 × 10^5^/mL, 1 mL) were seeded in confocal dishes and cultured with Dulbecco’s modified Eagle’s medium with 1% (v/v) penicillin/streptomycin and 10% (v/v) FBS for 24 h. Afterward, nanoparticles (PSMA-242, 2.4 ppm, 1 mL; PSMA-YI-1, 11.8 ppm, 1 mL) were added to the dishes, and the cells were cultured for another 16 h. Then, the cells were washed with medium three times and observed using a Nikon Inverted Multiphoton Microscope Eclipse (A1MP + Eclipse Ti-2E, Nikon instrument Inc., Japan) with a 40 × NA = 1.15 water-immersion objective.

### In vivo studies with a multiphoton microscope

For deep-tissue in vivo bioimaging, nanoparticles (PSMA-242, 72 ppm, 100 μL; PSMA-YI-1, 118 ppm, 100 μL) were injected intravenously into C57BL/6-C2J mice via the tail vein. During the injection, the ear was mounted on cover glasses and observed with a Nikon Inverted Multiphoton Microscope Eclipse (A1MP + Eclipse Ti-2E, Nikon instrument Inc., Japan) with a 40 × NA = 1.15 water-immersion objective. During the experiment, anesthesia was achieved by injecting Avertin intraperitoneally.

### In vivo PDT efficacy evaluation

The orthotopic bladder tumors were inoculated in the bladder of C57BL/6 mice with 1 × 10^6^ MB49 cells 10 days before starting treatment for a total of 2 doses of irradiation every 5 days. The mice were randomly divided into 2 groups (3 mice in each group) that received PBS or 120 ppm CPBA-conjugated PSMA-YI-1 NPs combined with 30 min of 660 nm laser irradiation (75 mW/cm^2^). The tumor volumes were estimated by a VisualSonics Vevo 770 High-Resolution Ultrasound Imaging System (Visual Sonics Inc, Toronto, Ontario, Canada) with a 25 MHz probe in B mode to identify the bladder tumor region. The tumor size changes were normalized to the initial tumor volume before treatment started and statistically analyzed by Prism (version 7; GraphPad, San Diego, CA, USA) with t test analysis (unpaired, one-tailed). The mice were anesthetized with 2% (v/v) isoflurane/O_2_ during all of the procedures. All animal studies were performed according to protocols approved by the Institutional Animal Care and Use Committee of National Cheng-Kung University.

### Histological examination

Histological analysis of mice tissues was performed after 20 days of treatment. Major organs (heart, liver, spleen, lungs, and kidneys) were collected and performed with hematoxylin and eosin stain. The H&E stained samples were then visualized by microscope (at 20× objective).

## Results and discussion

### Synthesis and characterization of the commercial dye-loaded PSMA NPs

**Fig. 2:**
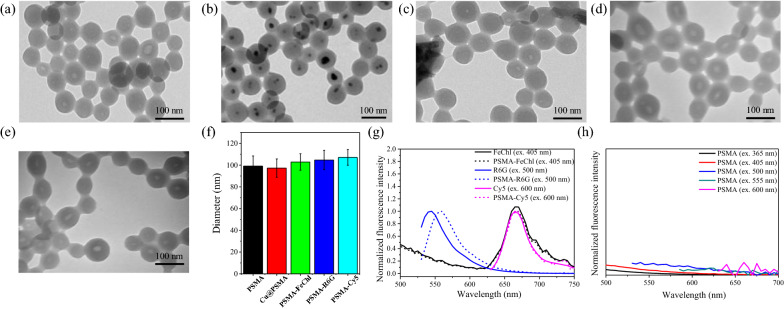
Transmission electron microscope (TEM) images of **a** PSMA, **b** Cu@PSMA, **c** PSMA-FeChl NPs, **d** PSMA-R6G NPs, and **e** PSMA-Cy5 NPs. **f** The corresponding DLS sizes from **a**–**e**. Fluorescent spectra of **g** FeChl-, R6G-, and Cy5-loaded PSMA NPs and **h** pure PSMA NPs

We prepared PSMA NPs with a diameter of 73.4 ± 11.9 nm (Fig. 2a). The single void (~ 17.1 ± 6.6 nm) in the central region of the PSMA nanoparticle was created by removing the metallic Cu core from the interior of the Cu@PSMA nanostructure (Fig. 2b) via the HCl etching process (Additional file 1: Fig. S1). Then, we loaded organic dyes with low molar mass (479.1–753.9 g/mol) into these nanosponges. The dye loading efficiency was ~ 3.7% for sodium iron chlorophyllin (FeChl), 50% for Cy5, and 26.5% for rhodamine 6 G (R6G). The TEM images (Fig. 2c–e) of their dye-loaded PSMA NPs showed a similar morphology and contrast as the empty PSMA carrier (Fig. 2a). Their particle sizes are almost the same as PSMA NPs. A slight increase in hydrodynamic diameters by DLS measurement compared with their solid form in TEM analysis was determined in Fig. 2f. This phenomenon can be explained by the positively charged Cy5 and R6G dyes, which prefer to load into/onto the negatively charged PSMA NPs (− 22.3 mV). These dye-loaded PSMA NPs exhibited fluorescence peak positions at 560 nm by R6G and ~ 670 nm by FeChl and Cy5 of visible emission signals similar to those of their free-form molecules in the solution (Fig. 2g). The FeChl- and Cy5-loaded PSMA NPs offered an extended emission to the NIR wavelength region. Without the loading of emitters, the PSMA NPs showed no significant emission signals upon excitation at 365–600 nm (Fig. 2h).

### Photophysical properties of the PSMA NPs loaded with OLED-, OPV-, and DSSC-functional molecules

**Fig. 3:**
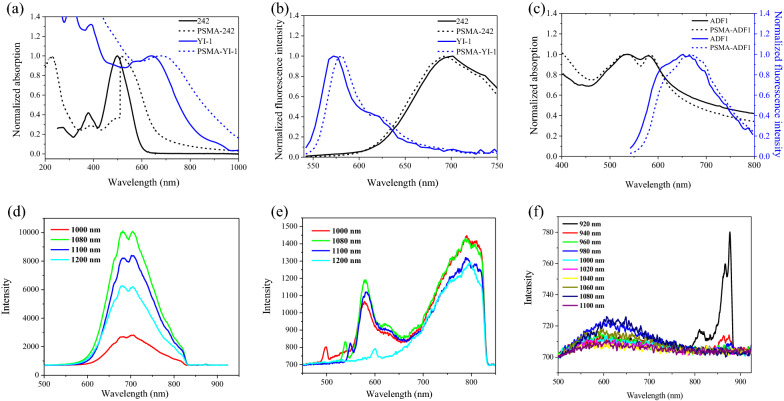
Absorption and multiphoton fluorescence spectra of PSMA loaded with dyes with high molar masses. **a** UV–Vis spectra and **b** single-photon fluorescent spectra (excited at 512 nm) of 242, YI-1 (solid line), PSMA-242, and PSMA-YI-1 NPs (dotted line). **c** Fluorescence spectra of ADF1 and PSMA-ADF1 NPs excited at 512 nm. Multiphoton fluorescence spectra of **d** PSMA-242, **e** PSMA-YI-1, and **f** PSMA-ADF1 NPs

Next, we examined the loading abilities of molecules with larger sizes and molar masses to demonstrate the versatility of the developed nanoformulation platform. We loaded PSMA NPs with an OPV-functional molecule, i.e., 242 (~ 2.5 nm in length; 772.4 g/mol), with a 4-dicyanomethylidenepyran-based core and 2-methylindoline-based donor moieties, and a DSSC-functional molecule, i.e., YI-1 (~ 3 nm in length; 1081.7 g/mol), with a donor-π bridge-acceptor structure using bis‐(4‐alkoxyphenyl)amine as the electron donor and indolium as the electron acceptor. In our previous report, we found that 242 was highly emissive in the deep red and NIR regions and that 242-based OLEDs exhibited high external quantum efficiency [44]. On the other hand, YI-1 exhibited both strong π-π transitions and intramolecular charge transfer (ICT) absorption bands ranging from the visible to NIR regions. Further details of the synthesis of 242 and YI-1 were described in the Additional file 1, including NMR and mass spectra results (Additional file 1: Scheme S1 and Figs. S2–S17). The loading efficiencies of PSMA NPs for 242 and YI-1 were both larger than 85% (Additional file 1: Fig. S18a). As shown in Fig. 3a, 242 exhibited a characteristic absorption band at 498 nm, which is redshifted by 12 nm (*J*-aggregation) [45] after loading into the PSMA NPs. On the other hand, a redshifted emission band for the PSMA-242 NPs was also observed when compared with that of 242 alone (Fig. 3b). A similar redshifting trend was found in the absorption and emission spectra for YI-1 and PSMA-YI-1 NPs (Fig. 3a and b).

In addition, we loaded three OLED-functional ADF-based molecules (Figs. 1a, 3c, and Additional file 1: Fig. S19) into PSMA NPs. The loading efficiencies of 85–88% and loading amount of 14–27 wt_[dye]_/wt_[PSMA]_% were accessed for ADF1 (~ 2.8 nm in length; 954.5 g/mol), ADF2 (~ 2.1 nm in length; 754.5 g/mol), and ADF3 (~ 1.8 nm in length; 506.2 g/mol) (Additional file 1: Fig. S18b). In terms of their fluorescence properties, ADF1-, ADF2-, and ADF3-loaded PSMA NPs exhibited a red emission band at 660 nm, green emission band at 515 nm, and blue emission band at 435 nm, respectively.

The water is a poor solvent for the 242, YI-1, and ADF-series dyes, which possess more hydrophobic properties than the ionic R6G, FeChl, and Cy5. Therefore, the diffusion of these hydrophobic dyes into the polystyrene blocks of PSMA NPs would be preferred and may decrease the free energy [46–48] in the system. This could be interpreted that the intermolecular encapsulation process can minimize the surface tension by preventing these hydrophobic dyes from precipitation in the poor solvent. The enhanced loading of hydrophobic drugs into the hydrophobic chain segments of the PSMA micelle can be found in the literature [49–51]. Nevertheless, the hydrophilic dye molecules can interact with the carboxylate groups at the surface PSMA via electrostatic interaction. Still, the solvation effect existed for ionic compounds in the aqueous environment [16, 19, 36]. The contradictory driving force resulted in a less loading amount for the ionic compounds (Additional file 1: Fig. S18).

### Analysis of multiphoton properties of dye-loaded nanoparticles

For deep-tissue imaging applications, we further investigated the multiphoton spectra of PSMA-242, PSMA-YI-1, and PSMA-ADF1 NPs (Fig. 3d–f). The PSMA-242 NPs exhibited two-photon fluorescence peaks at approximately 700 nm under varying excitation wavelengths from 1000 to 1200 nm (Fig. 3d). Interestingly, the PSMA-YI-1 NPs have two fluorescence peaks at 600 and 800 nm (Fig. 3e). Both PSMA-242 and PSMA-YI-1 exhibited NIR-excited multiphoton NIR-fluorescence emission features. In contrast, PSMA-ADF1 NPs exhibit weak luminescence at ~ 605 nm when excited at 920 nm (Fig. 3f). We also loaded a new donor–acceptor–donor (D–A–D)-configured fluorescent dye 4,8-bis(4-(di-p-tolylamino)phenyl)-6-(2-ethylhexyl)-5H-[1,2,5]thiadiazolo[3,4-f]isoindole-5,7(6H)-dione (DTDPTID) (Fig. 1a, Additional file 1: Scheme S2, Figs. S20 and S21) into PSMA NPs. This resulting product performed a strong and primary emission between 650 and 700 nm (Additional file 1: Fig. S22). At a threefold concentration of PSMA-242, a sixfold two-photon fluorescence intensity was obtained. We measured the two-photon absorption (TPA) coefficient *β* of these chromophore centers (Additional file 1: Fig. S23). We found the *β* and *σ* of DTDPTID are 17–220 times larger than other organic dyes (Additional file 1: Table S1). The high TPA cross section of DTDPTID could be attributed to its multipolar D-A-D structure [52]. After loading into PSMA NPs, the highly condensed DTDPTID molecules in the PSMA NPs exhibited a relatively low *β* (Additional file 1: Table S1) that could be attributed to the nano-aggregation effects of light-scattering phenomenon and the enhanced non-radiative decay caused by the interchromophoric [53] and DTDPTID-PSMA interactions.

### Surface characterization of dye-loaded PSMA NPs

Based on the FT-IR spectrum analysis (Additional file 1: Fig. S24), the typical absorption bands of 242, YI-1, and ADF1 overlapped with those of the PSMA NPs at 1720–1760 cm^−1^ resulting from C=O groups, 1601–1584 cm^−1^ resulting from C=C stretching within the ring of styrene, 1450 cm^−1^ resulting from _νasym_COO^−^ mode and 1340 cm^−1^ resulting from _νsym_COO^−^ mode. The IR spectra of the dye-loaded PSMA NPs and the bare PSMA NPs are almost the same. The zeta potential of each of the dye-loaded PSMA NPs was approximately − 20 mV (Additional file 1: Fig. S25), indicating a negatively charged surface with carboxylate terminals exposed to water. This surface charge property is beneficial for the good dispersion of PSMA NPs in aqueous solutions.

The transmission electron microscopy (TEM) images revealed that PSMA NPs showed increased contrast after loading with photosensitive molecules with larger size and higher hydrophobicity (Additional file 1: Fig. S26). The increased contrast (Additional file 1: Fig. S26f) can be attributed to the high loading efficiencies of dye molecules into organic carriers [54, 55]. The loading efficiency and loading amount per PSMA weight were measured as 85% and 27 wt_[dye]_/wt_[PSMA]_% for ADF1, 99% and 24 wt_[dye]_/wt_[PSMA]_% for 242, and 87% and 36 wt_[dye]_/wt_[PSMA]_ % for YI-1 (Additional file 1: Fig. S18). These aforementioned amounts displayed a little more adsorption of dye molecules than the assembled PSMA-dye micelles [49–51]. On the other hand, the resulting particle size was slightly increased by 1.5–7.8 nm, and some particles had enlarged void sizes in the interior (Additional file 1: Table S2). The negative stain images of dye-loaded PSMA NPs were also performed. As shown in Additional file 1: Fig. S27, there is no additional organic gap or irregular small dye aggregates appeared between the uranyl acetate staining materials and PSMA NPs, suggesting that the sponge-like swelling nature of the PSMA NPs allows the penetration of these photosensitive molecules into the interspace of NPs (Fig. 1b) instead of just adsorbed on the surface of NPs. Moreover, the potential leakage of the dyes was below 6.25% after two days of aging (Additional file 1: Fig. S28a). Additional file 1: Fig. S28b shows the DLS measurement to determine the hydrodynamic diameters of dye-loaded PSMA NPs increased by 23–29 nm compared with their dry form in the TEM images. As expected, the hydrophilic domains in the PSMA structure could be swelled in water leading to particle volume expansion [50, 54, 56]. No significant size changes were found in physiological buffer solution, indicating the high stability of the PSMA NPs carried dyes.

Without help from carriers, hydrophobic 242, YI-1, and ADF1 photosensitive molecules will aggregate in the blood [57] and undergo undesired rapid clearance by the endothelial reticular system. One common solution for this is to carry these chromophores in a polymer through a phospholipid packaging method to form encapsulated NPs [58, 59]. The disadvantage of this method is a low loading rate. In addition, there are concerns about the undesired release of the dyes. Although synthetic methods, such as polymer grafting, AIE self-assembly, and coprecipitation methods, can be used to assemble light-emitting dye nanoparticles [18, 20–23], the synthesis parameters of these approaches still require optimization. In our current work, the assembly of PSMA NPs is achieved by a straightforward strategy (Fig. 1b) to prepare organic nanoemitters by a simply physical mixture process to load small commercially available dyes and newly synthesized large dyes without tedious synthetic procedures (e.g., ultrasonic probe to create microemulsion and time-consuming solvent evaporation process) [20, 55].

### Cell viability of dye-loaded PSMA NPs

**Fig. 4:**
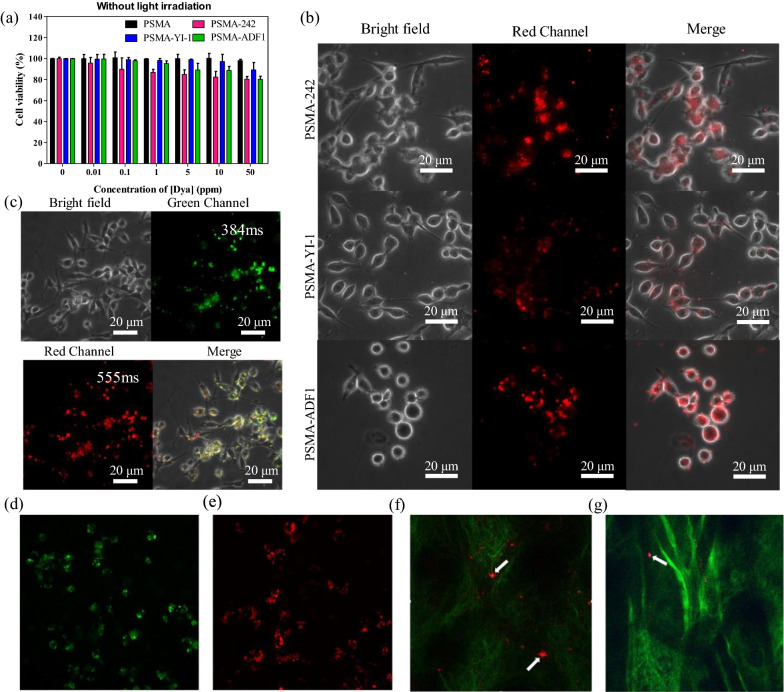
**a** Biocompatibility of PSMA NPs loaded with different dyes. Fluorescent images of cells labeled with **b** PSMA-242, YI-1, ADF1 single staining or **c** PSMA-(ADF1 + ADF2) double staining and tracked by fluorescence microscopy. Scale bars = 20 μm. Multiphoton imaging of **d** PSMA-242 and **e** PSMA-YI-1 excited at 1080 nm. In vivo two-photon fluorescence (red color) and second harmonic generation (green color) imaging of dye-loaded PSMA NPs circulated in the blood vessels of a mouse ear. **f** PSMA-242; **g** PSMA-YI-1; excitation wavelength: 1080 nm. Fields of view: 317 × 317 μm

The cytotoxicity of these 242-, YI-1-, and ADF1-loaded PSMA NPs was further evaluated by MTT assay. The cell viability was over 80.2% throughout the concentration range of 0 ppm_[dye]_–50 ppm_[dye]_ for all samples incubated with MB49 cancer cells (1 day), demonstrating the reduced cytotoxicity of these molecules (Fig. 4a) compared to that of free dye molecules (Additional file 1: Fig. S28c). Their low cytotoxicity implies the low leakage of dyes (Additional file 1: Fig. S28a). Direct treatment of these dyes at the same released concentration did not cause cell toxicity (Additional file 1: Fig. S28c). Using JC-1 staining, we further confirmed the unchanged bioactivity of the mitochondria in MB49 cells after treatment with dye-loaded PSMA NPs (Additional file 1: Fig. S29).

### Single-photon cellular imaging of dye-loaded PSMA NPs

After observing the red-NIR emission characteristics of the dye-loaded PSMA NPs (Fig. 3b and c), we treated MB49 cells with PSMA NPs loaded with 242, YI-1, and ADF1 and explored their potential applications as biological probes. Fluorescence images (Fig. 4b) showed that all three nanoemitters appeared red within MB49 cells after 24 h of coculture. The 530–550 nm excited red fluorescence was detected by employing a longpass filter (Olympus, UFGW) with an edge wavelength of 575 nm. Based on the confocal microscopy image, these red fluorescent dots of PSMA-ADF1 (as a modeling nanoemitter) in cells were overlaid with the green fluorescence of the lysosome tracker, which is represented as yellow (Additional file 1: Fig. S30a).

The wavelength tunability of the labeling can be easily achieved by choosing the emitter color of fluorescence. As a demonstration, we prepared ADF1-, ADF-2-, and ADF3-loaded PSMA NPs that exhibited individual red, green, and blue fluorescence properties to stain MB49 cancer cells after 24 h of incubation (Additional file 1: Fig. S30b). Then, we codelivered both ADF1- and ADF2-loaded PSMA NPs to MB49 cancer cells. A fluorescence microscope could separately visualize the emission signals from the green and red emitters in the MB49 cells (Fig. 4c). In the merged image, most of the emission color manifested as yellow due to the colocalization of the ADF1- and ADF2-loaded PSMA NPs, and combined with the confocal microscopy images of PSMA-ADF1 and lysosomes, it showed a colocalized position (Additional file 1: Fig. S30a), suggesting the internalization of ADF1- and ADF2-loaded PSMA NPs in the endolysosomes. We believe that these nanoemitters, combining different colors of dye molecules with strong fluorescent properties, can replace small molecular dyes, thereby allowing the development of biological immunofluorescence staining technology with low concentrations of biomolecules.

### Multiphoton bioimaging in vitro and in vivo

To demonstrate the multiphoton contrasts of dye-loaded PSMA NPs in biomedical imaging, we treated MDA-MB-231 cells with PSMA-242 and PSMA-YI-1 NPs for 16 h. The cells were observed using an inverted multiphoton microscope (Nikon, A1 MP+ with Ti2 E). The multiphoton fluorescence signals from PSMA-242 (Fig. 4d) and PSMA-YI-1 NPs (Fig. 4e) were mainly distributed in the cytoplasm, demonstrating that the nanoparticles were biocompatible and able to enter cells through endocytosis. To demonstrate in vivo deep-tissue imaging, we tail-vein injected the PSMA-242 and PSMA-YI-1 NPs to circulate them in the blood of mice and successfully observed their presence in the blood vessels of mouse ears (Fig. 4f and g). These NPs were able to circulate to the ear (white arrows) after tail-vein injection (see Additional file 2: Movie S1 and Additional file 3: Movie S2). These features validated that dye-loaded PSMA NPs can serve as NIR-active multiphoton contrast agents in deep-tissue biomedical imaging.

As mentioned above, PSMA-DTDPTID NPs (Additional file 1: Figs. S22 and S23) exhibited a better two-photon quantum yield due to its D–A–D-configured molecular structure, which can generate efficient charge transfer interactions [52] and has a more promising detection ability for highly sensitive biolabeling in vivo. To answer our proposed statement, we additionally performed the two-photon in vivo angiography with PSMA-DTDPTID NPs. As we expected, the imaging contrast has significant improvement (Additional file 1: Fig. S31) than PSMA-242 and PSMA-YI-1 NPs.

### NIR-based PDT

**Fig. 5:**
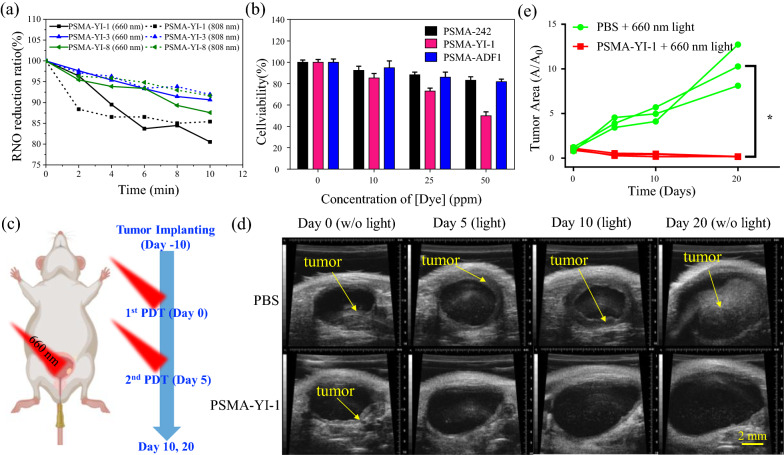
**a** Singlet oxygen generation of PSMA NPs loaded with YI series dyes by RNO/imidazole assay under 10 min of 660 (solid lines) and 808 (dotted lines) nm laser irradiation (75 mW/cm^2^ and 0.9 W/cm^2^, respectively). **b** PDT efficacy of PSMA-242, PSMA-YI-1, and PSMA-ADF1 NPs in MB49 cancer cells under 808 nm laser irradiation (0.9 W/cm^2^) for 10 min. **c** Schematic of the in vivo treatment process. **d** Ultrasound images of a mouse bladder with tumors before and after treatment with PBS and CPBA-conjugated PSMA-YI-1 NPs combined with 30 min of 660 nm laser irradiation (75 mW/cm^2^). Scale bar = 2 mm. **e** Tumor area changes quantified from **d**. (*p < 0.05, compared with PBS group)

Finally, we examined the photodriven toxicity of the PMSA-YI-1 NPs by using a RNO/imidazole indicator [60]. The stability test revealed that the loading of YI-1 into PSMA NPs increased their optical stability in a physiological buffer (PBS, pH = 4 and 7) environment (Additional file 1: Fig. S32) compared to that of the dye molecules alone under the same conditions. Considering the broadened absorption range of the PMSA-YI-1 NPs (Fig. 3a), we utilized a 660 nm laser and 808 nm laser (Fig. 5a) to access the energy transfer from the triplet state of the PMSA-YI-1 NPs to ^3^O_2_, leading to the production of ^1^O_2_ species by the intersystem crossing pathway. We observed a decrease in the absorbance at 440 nm by the RNO molecule as a function of irradiation at 660 nm (75 mW/cm^2^) and 808 nm (0.9 W/cm^2^), indicating the successful production of ^1^O_2_ molecules after the light excitation of the PMSA-YI-1 NPs. Approximately 40.5% at 660 nm and 54.2% at 808 nm of PDT activity decay were measured for the PMSA-YI-1 NPs compared to the performance of ^1^O_2_ species generation from the free YI-1 photosensitive molecules (Additional file 1: Fig. S33). This decreased efficiency could be attributed to the tremendous adsorption of the YI-1 molecules and thus caused energy compulsion via intermolecular interactions [57]. The PSMA NPs did not contribute to the production of ^1^O_2_ molecules when irradiated with 808 nm laser light.

On the other hand, we found that the NIR-response of ^1^O_2_ produced by the PMSA-YI-1 NPs was larger than that of the other DSSC-based analog molecules (Additional file 1: Fig. S33). This result can be attributed to the strong absorbance of YI-1 in the NIR region (ε = 23,000 M/cm at 808 nm), which increases the probability of YI-1 intersystem crossing to its triplet excitation state such that more singlet oxygen was generated. In addition, such a nonplanar extended π-conjugated system also results in a small energy gap of YI-1 (1.29 eV) that could facilitate energy transfer from the sensitizer to ground-state oxygen (^3^O_2_) of which the ^1^Δ_g_ state is 0.98 eV. In previous study, Fu and co-workers demonstrated the distorted π-conjugated structures of triply linked bay-fused diperylene bisimides leads to the breakdown of the σ-π orbital separation and thus enhances the spin-orbital coupling required of intersystem crossing [61]. Indeed, as illustrated in the left of Fig. 1c, the non-emissive property of YI-1 also suggests a decreased rate of fluorescence decay from the excited state that improves the rate of ISC as well as the energy transfer from YI-1 to ^3^O_2_. Similar phenomenon was reported in several previous works using NIR dye as the photosensitizer to study the generation of singlet oxygen [61–63].

Because of the exposure of the carboxylate groups in the dye-loaded PSMA NPs, 4-carboxyphenylboronic acid (CPBA) was conjugated to the nanoemitters to enhance targeted delivery to the glycoprotein receptors of the MB49 cells. Figure 5b shows an ~ 50% decrease in cell viability in PSAM-YI-1 NP-treated MB49 cells by 808 nm laser irradiation (0.9 W/cm^2^, 10 min). On the other hand, we found that the culture medium was maintained below 38 °C during the same light incident (Additional file 1: Fig. S34), indicating successful PDT-elicited phototoxicity and low side effects by heating. By screening with the other dye-loaded PSMA NPs, they presented NIR-photoactive ROS (e.g., PDT) that were negligible with regard to cells (Additional file 1: Fig. S35).

To suggest the concept that NIR-PDT treatment is promising for the depletion of solid tumors, we further investigated the in vivo PDT efficacy of PSMA-YI-1 NPs in an orthotopic bladder cancer mouse model [39]. Mice were first intrabladder inoculated with tumors of 1 × 10^6^ MB49 cells at 10 days before starting treatment and received irradiation every 5 days (Fig. 4c). The mice receiving 120 ppm CPBA-conjugated PSMA-YI-1 NPs combined with 30 min of 660 nm laser irradiation (75 mW/cm^2^) showed a significant tumor volume decrease after 2 rounds of PDT treatment according to ultrasound imaging observations, while the tumor size in the PBS-treated group continuously increased and eventually occupied almost the entire bladder cavity by Day 20 (Fig. 5d and e). The subsequent pathology assessment of mouse organs through H&E stain revealed that no obvious damage or inflammation in heart, liver, spleen, lung and kidney was observed in PSMA-YI-1 NPs-treated mice (Additional file 1: Fig. S36), demonstrated no systemic toxicity of PSMA-YI-1 NPs on mice model. Our preliminary results suggested that the PSMA-YI-1 NPs not only possess in vitro PDT properties but also exhibit successful in vivo PDT efficacy.

## Conclusions

Based on the above TEM and optical analysis results, we verified that the PSMA structure was applicable not only for the loading of commercial dyes with small molar masses but also for the facile enhancement of the loading of photosensitive molecules with molar masses up to 1081.7 g/mol and molecular lengths of approximately 3 nm. Our results from synthetic design, fluorescence spectroscopy, biomedical imaging, and photodynamic therapy validated that the delicate PSMA nanosponge can serve as a biocompatible platform for assembling organic nanoemitters. Due to the flexibility of the synthetic strategy, we produced nanoparticles with multiple emission wavelengths ranging from the visible to NIR regions. These organic nanoemitters exhibited single-photon and multiphoton NIR luminescence for bioimaging applications and phototherapeutic activation under NIR stimulation. Based on the successful demonstration of the potential targeted-PDT of PSMA-YI-1 NPs in bladder cancer cells, the additional loading of chemo-drugs into the PSMA nanocarriers may result in better anticancer efficacy with lower dose administration for future chemotherapeutic strategies.

## Supplementary Information

**Additional file 1. **Additional Materials and methods, additional Schemes S1, S2, additional Figures S1–S36, additional Tables S1, S2.

**Additional file 2.** In vivo two-photon fluorescence video of PSMA-242 NPs circulating in mouse ear vessels.

**Additional file 3.** In vivo two-photon fluorescence video of PSMA-YI-1 NPs circulating in mouse ear vessels.

## Data Availability

All data generated or analyzed during this study are included in this published article.
