# Supplementary material for: A universal strategy for the fabrication of single-photon and multiphoton NIR nanoparticles by loading organic dyes into water-soluble polymer nanosponges
Source: J Nanobiotechnology. 2022 Jul 6;20:311. doi: 10.1186/s12951-022-01515-5 (PMC9258130; doi:10.1186/s12951-022-01515-5)
Supplement: Supplementary file 1 — Additional file 1. Additional Materials and methods, additional Schemes S1, S2, additional Figures S1–S36, additional Tables S1, S2. [file 12951_2022_1515_MOESM1_ESM.docx]

Supporting Information

A universal strategy for the fabrication of single-photon and multiphoton NIR nanoparticles by loading organic dyes into water-soluble polymer nanosponges

Li-Xing Yang,^1^† Yu-Cheng Liu,^2^† Chang-Hui Cho,^3^† Yi-Rou Chen,^4^† Chan-Shan Yang,^5^† Yin-Lin Lu,^2^ Zhiming Zhang,^2^ Yi-Tseng Tsai,^1^ Yu-Cheng Chin,^1^ Jiashing Yu,^6^ Hsiu-Min Pan,^4^ Wei-Rou Jiang,^1^ Zi-Chun Chia,^1^ Wei-Shiang Huang,^1^ Yu-Lin Chiu,^3^ Chun-Kai Sun,^6^ Yu-Ting Huang,^7^ Li-Ming Chen,^7^ Ken-Tsung Wong,^7^ Han-Min Huang,^5^ Chih-Hsin Chen,^4*^ Yuan Jay Chang,^3*^ Chih-Chia Huang ^1,8*^ and Tzu-Ming Liu ^2*^

1. Department of Photonics, National Cheng Kung University, Tainan 70101, Taiwan.

2. Institute of Translational Medicine, Faculty of Health Sciences & Ministry of Education Frontiers Science Center for Precision Oncology, University of Macau, Macao SAR 999078, China.

3. Department of Chemistry, Tunghai University, Taichung 40704, Taiwan.

4. Department of Chemistry, Tamkang University, New Taipei City 25137, Taiwan.

5. Institute and Undergraduate Program of Electro-Optical Engineering, National Taiwan Normal University, Taipei 11677, Taiwan

6. Department of Chemical Engineering, National Taiwan University, Taipei 106, Taiwan.

7. Department of Chemistry, National Taiwan University, Taipei 10617, Taiwan.

8. Center of Applied Nanomedicine, National Cheng Kung University, Tainan 70101, Taiwan.

Prof. Chih-Hsin Chen

E-mail: chc@mail.tku.edu.tw

Prof. Yuan Jay Chang

E-mail: jaychang@thu.edu.tw

Prof. Chih-Chia Huang

E-mail: c2huang@mail.ncku.edu.tw, huang.chihchia@gmail.com

Prof. Tzu-Ming Liu

E-mail: tmliu@umac.mo

† L.-X. Yang, Y.-C. Liu, C.-H. Cho, Y.-R, Chen and H.-M. Huang contributed equally to this work.

**Materials and Methods**

***Synthesis of the organic chromophores***

**General Aspects.**

All reactions were conducted under a nitrogen atmosphere unless otherwise noted. All solvents were dried over appropriate drying agents and distilled before use. Commercially available reagents were used without further purification. Reactions were monitored by pre-coated TLC plates (0.20 mm with fluorescent indicator F_254_). Mass spectra were obtained on a THERMO Q Exactive Plus instrument operating in electrospray ionization (ESI) mode. ^1^H and ^13^C NMR spectra were recorded on a Bruker Ascend NMR 400 MHz. ^1^H NMR spectra were obtained in CDCl_3_ and calibrated using residual CHCl_3_ (*δ* = 7.26 ppm) as an internal reference. UV-Vis absorption and PL spectra were measured by an Edinburgh FS5 Spectrofluorometer. Oxidation potentials were measured through cyclic voltammetry using a CHI 620 analyzer. All measurements were performed in methylene chloride solutions containing 0.1 M tetrabutylammonium hexafluorophosphate as the supporting electrolyte under ambient conditions after purging for 10 min with N_2_. Furthermore, the conventional three-electrode configuration was employed, comprising a glassy carbon working electrode, a platinum counter electrode, and an Ag/Ag^+^ reference electrode calibrated using ferrocene/ferrocenium as an internal reference. Mass spectra were recorded using a JEOL JMS-700 double-focusing mass spectrometer. Thermal analysis was performed using a Mettler-Toledo, 2-HT thermogravimetric analyzer under nitrogen at a heating rate of 10 °C min^-1^.

The starting materials, such as 2-methylindoline, 1-bromo-4-(*tert*-butyl)benzene, POCl_3_, piperidine, trifluoroacetic acid (TFA), *N*-bromosuccinimide (NBS), phenylboronic acid, trimethylsilylacethylene, copper(I) iodide, zinc, diisopropylamine (*i*-Pr_2_NH), bromine (Br_2_), 2,7-dibromo-9*H*-fluorene, 1-bromohexane, bis(4-(hexyloxy)phenyl)amine, 5-bromothiophene-2-carbaldehyde, potassium *tert*-butoxide, potassium carbonate, Pd(OAc)_2_, Pd(dppf)Cl_2_, Pd(PPh_3_)_4_, dppf, were purchased commercially and used without further purification, unless otherwise stated. Compounds 2-(2,6-dimethyl-4H-pyran-4-ylidene)malononitrile and 5-carboxy-2,3,3-trimethyl-1-hexyl-3*H*-indolium iodide were synthesized according to literature reported procedures (see below)[33] and were characterized by ^1^H, ^13^C NMR spectroscopy techniques as described below, and the data were matched with the literature citations.

**Synthesis: *Experimental procedure***

**Scheme S1**: Synthesis of the **242,** **ADF1~3** and **YI-1** dyes. Reagents: (i) Pd(OAc)_2_, dppf, Na*^t^*OBu, 4-bromo-*tert*-butylbenzene, toluene, 90 °C; (ii) POCl_3_ , DMF, 90 °C; (iii) 2-(2,6-dimethyl-4H-pyran-4-ylidene)malononitrile, piperidine, MeCN, reflux; (iv) TFA, *t*-BuOH, 90℃; (v) NBS, CH_2_Cl_2_, r.t.; (vi) phenylboronic acid, Pd(PPh_3_)_4_, K_2_CO_3,_ toluene / THF=2/1, reflux.; (vii) trimethylsilylacethylene, CuI, Pd(PPh_3_)_4_, *i*-Pr_2_NH / THF=1/3, 80℃; (viii) K_2_CO_3_, MeOH / THF=1/1, r.t.; then **4**, CuI, Pd(PPh_3_)_4_, *i*-Pr_2_NH / THF=1/3, 80℃; (ix) Zn, HOAc/HCl=1/1, 90℃; (x) Br_2_, CH_2_Cl_2_, 0℃~r.t.; (xi) 1-bromohexane, KO*^t^*Bu, THF, r.t.; (xii) bis(4-(hexyloxy)phenyl)amine, Pd(OAc)_2_, dppf, NaO*^t^*Bu, toluene, reflux.; (xiii) 5-bromothiophene-2-carbaldehyde, Pd(PPh_3_)_4_, K_2_CO_3,_ toluene / THF=2/1, reflux.; (xiv) 5-carboxy-2,3,3-trimethyl-1-hexyl-3*H*-indolium iodide, piperidine, acetonitrile, reflux.

*1-(4-tert-Butylphenyl)-2-methylindoline* **(1)**

A stirred mixture of Pd(OAc)_2_ (38 mg, 0.17 mmol), dppf (148 mg, 0.27 mmol), and 4-bromo-1-*tert*-butylbenzene (2.46 g, 11.6 mmol) in toluene was placed in a three-necked flask under a nitrogen atmosphere and was stirred at 100 °C. After 15 min, to it was added 2-methylindoline (1.54 g, 11.6 mmol) and NaO*^t^*Bu (1.67 g, 17.4 mmole) under a nitrogen atmosphere and the mixture was stirred at 100 °C for 24 h. After cooling, the reaction was quenched by adding water, then was extracted with ethyl acetate. The organic layer was dried over anhydrous MgSO_4_ and evaporated under a vacuum. The products were purified by silica gel column chromatography eluted with CH_2_Cl_2_/hexane (1/3). The yellow liquid was isolated in 85% yield (2.61 g, 9.86 mmol). Spectroscopic data of **1**: *δ*_H_ (400 MHz, CDCl_3_) 7.36 (d, 2H, *J* = 8.6 Hz), 7.17 (d, 2H, *J* = 8.6 Hz), 7.10 (d, 1H, *J* = 7.2 Hz), 6.99 (t, 1H, *J* = 7.6 Hz), 6.76 (d, 1H, *J* = 7.9 Hz), 6.68 (t, 1H, *J* = 7.3 Hz), 4.29-4.35 (m, 1H), 3.29 (dd, 1H, *J* = 15.3, 7.7 Hz), 2.73 (dd, 1H, *J* = 15.3, 7.7 Hz), 1.33 (s, 9H), 1.30 (d, 3H, *J* = 6.0 Hz); *δ*_C_ (100 MHz, CDCl_3_) 149.1, 145.6, 140.8, 129.3, 127.0, 125.9, 124.6, 121.4, 118.2, 108.1, 37.1, 34.2, 31.4, 20.1; *m/z* (FAB) 265.1824 (M^+^. C_19_H_23_N requires 265.1830).

*1-(4-tert-Butylphenyl)-2-methylindoline-5-carbaldehyde* **(2)**

A stirred mixture of POCl_3_ (1.2 mL, 12.4 mmol), and N,N-dimethylformamide (DMF) (0.9 mL, 12.4 mmol) was placed in a three-necked flask under N_2_ in an ice bath for 30 min. Compound **1** (2.75 g, 10.4 mmol) was dissolved in 15 mL DMF, and was added dropwise into the mixture. The mixture was stirred at 90 °C for 12 h. After cooling, the reaction was quenched by adding aqueous CH_3_COONa, and was extracted with EA. The organic layer was dried over anhydrous MgSO_4_ and evaporated under a vacuum. The product was purified by silica gel column chromatography with EA/hexane (1/19) as eluent. Yellow solids of **2** were obtained in 90% yield (2.74 g, 9.36 mmol). ^1^H NMR (400 MHz, 298 K, CDCl_3_) *δ* 8.27 (d, *J* = 8.2 Hz, 1H), 8.17 (d, *J* = 8.4 Hz, 1H), 8.14 (d, *J* = 7.7 Hz, 3H), 8.11 (d, *J* = 7.7 Hz, 1H), 7.98 (d, *J* = 8.0 Hz, 2H), 7.85 (t, *J* = 7.7 Hz, 1H), 7.78 (d, *J* = 7.9 Hz, 2H), 7.68 (d, *J* = 8.1 Hz, 4H), 7.56 (d, *J* = 8.3 Hz, 4H), 7.52 (d, *J* = 7.5 Hz, 1H), 7.42 (dd, *J* = 11.4, 6.4 Hz, 8H), 7.38 (s, 2H), 7.29 (dd, *J* = 9.6, 4.1 Hz, 4H), 7.17 (t, *J* = 7.6 Hz, 1H), 6.80 (d, *J* = 8.0 Hz, 1H). ^13^C NMR (100 MHz, 298 K, CDCl_3_) *δ* 148.7, 140.8, 140.19, 139.9, 139.0, 138.2, 136.9, 135.2, 130.6, 129.9, 129.5, 128.8, 128.4, 128.3, 127.6, 127.2, 125.9, 124.3, 123.4, 121.2, 120.3, 120.0, 109.8. HRMS (*m/z*, FAB) [M]^+^ calcd for C_62_H_39_N_2_O: 826.2934, found 827.3057.

*2-(2,6-bis((E)-2-(1-(4-(tert-butyl)phenyl)-2-methylindolin-5-yl)vinyl)-4H-pyran-4-ylidene)malononitrile* (**242**)

A mixture of **2** (2.92 g, 10 mmol), 2-(2,6-dimethyl-4H-pyran-4-ylidene) malononitrile (860 mg, 5.0 mmol), and piperidine (1.1 mL, 11 mmol) was placed in a three-necked flask containing dry acetonitrile (30 mL) under N_2_. The resulted mixture was heated to reflux for 24 hours. After cooling, the reaction was quenched by pouring it into water and it was extracted with CH_2_Cl_2_. The combined organic layer was dried over anhydrous MgSO_4_ and evaporated under a vacuum. The mixture was purified by silica gel column chromatography with CH_2_Cl_2_ as eluent. The red solid of **242** was collected in 82% yield (6.33 g, 8.2 mmol). Mp: 231~233℃. ^1^H NMR (400 MHz, 298 K, CDCl_3_) *δ* 7.35-7.41 (m, 8H), 7.16-7.20 (m, 6H), 6.70 (d, *J* = 8.3 Hz, 2H), 6.50 (d, *J* = 2.0 Hz, 2H), 6.46 (d, *J* = 15.7 Hz, 2H), 4.45-4.49 (m, 2H), 3.35 (dd, *J* = 15.8, 7.4 Hz, 2H), 2.80 (dd, *J* = 15.8, 7.4 Hz, 2H), 1.33 (s, 18H), 1.30 (t, *J* = 6.15 Hz, 6H). ^13^C NMR (100 MHz, 298 K, CDCl_3_) *δ* 159.2, 156.0, 151.5, 147.2, 138.8, 138.2, 130.4, 129.8, 126.5, 124.9, 123.5, 122.4, 116.2, 113.0, 107.4, 105.3, 60.2, 56.1, 36.4, 34.4, 31.3, 20.1. HRMS (m/z, FAB) [M]^+^ calcd for C_50_H_50_N_4_O: 722.3985, found 722.3982.

*2,6-di-tert-butylanthracene* (**3**)

A stirred mixture of anthracene (10 g, 61 mmol), trifluoroacetic acid (TFA) (70 mL), and was placed in a three-necked flask under N_2_ at 90℃ for 16 h. After cooling, the reaction was quenched by adding aqueous NaHCO_3_, and was extracted with methylene chloride. The organic layer was dried over anhydrous MgSO_4_ and evaporated under a vacuum. The product was washed by hexane. White solids of **3** were obtained in 75% yield (13.27 g, 45.75 mmol). ^1^H NMR (400 MHz, 298 K, CDCl_3_) *δ* 8.33 (s, 2H), 7.93 (d, 2H, *J* = 9.0 Hz), 7.87 (d, 2H, *J* = 1.5 Hz), 7.55 (dd, 2H, *J* = 9.0 Hz) ^13^C NMR (100 MHz, 298 K, CDCl_3_) *δ* 147.3, 131.6, 130.5, 127.7, 125.4, 124.8, 122.3, 34.9, 31.0.

*9,10-dibromo-2,6-di-tert-butylanthracene* (**4**)

A mixture of **3** (5 g, 17 mmol), and N-Bromosuccinimide (NBS) (860 mg, 5.0 mmol) was placed in a three-necked flask containing methylene chloride (150 mL) under N_2_. The resulted mixture was stirred at room temperature. After 8 h, the reaction was extracted with methylene chloride. The combined organic layer was dried over anhydrous MgSO_4_ and evaporated under a vacuum. The mixture was purified by silica gel column chromatography with hexane as eluent. The yellow solid of **4** was collected in 95% yield (7.2 g, 16.15 mmol). ^1^H NMR (400 MHz, 298 K, CDCl_3_) *δ* 8.51 (d, 2H, *J* = 9.3 Hz), 8.46(d, 2H, *J* = 1.8 Hz), 7.72 (dd, 2H, *J* = 9.3 Hz), 1.45 (s, 18H). ^13^C NMR (100 MHz, 298 K, CDCl_3_) *δ* 150.0, 130.8, 130.1, 128.2, 127.1, 123.2, 122.8, 35.5, 31.1.

*9-bromo-2,6-di-tert-butyl-10-phenylanthracene* (**5**)

A mixture of **4** (5 g, 11 mmol), phenylboronic acid (860 mg, 5.0 mmol), Pd(PPh_3_)_4_ (510 mg, 0.33 mmol), K_2_CO_3_ (2M, 5 mL) was placed in a three-necked flask containing THF (25 mL)/toluene (50 mL) under N_2_. The resulted mixture was stirred at 90℃. After 12 h, the reaction was extracted with methylene chloride. The combined organic layer was dried over anhydrous MgSO_4_ and evaporated under a vacuum. The mixture was purified by silica gel column chromatography with hexane as eluent. The yellow solid of **5** was collected in 75% yield (3.66 g, 8.25 mmol). ^1^H NMR (400 MHz, 298 K, CDCl_3_) *δ* 8.54 (d, 1H, *J* = 9.3 Hz), 8.49(d, 1H, *J* = 1.5 Hz), 7.68 (dd, 1H, *J* = 9.3 Hz) , 7.62~7.54 (m, 5H) , 7.48~7.40 (m, 3H) , 1.48 (s, 9H) , 1.29 (s, 9H). ^13^C NMR (100 MHz, 298 K, CDCl_3_) *δ* 149.3, 147.7, 138.9, 131.3, 130.9, 130.0, 129.9, 128.5, 127.8, 127.7, 127.3, 126.5, 125.2, 122.4, 122.3, 121.7, 35.5, 35.1, 31.2, 30.1.

*9-bromo-2,6-di-tert-butyl-10-phenylanthracene* (**6**)

A mixture of **5** (3 g, 6.74 mmol), trimethylsilylacethylene (1.44 mL, 10.11 mmol), CuI (38 mg, 0.2 mmol), Pd(PPh_3_)_4_ (233 mg, 0.2 mmol) was placed in a three-necked flask containing *i*-Pr_2_NH(5 mL)/toluene (15 mL) under N_2_. The resulted mixture was stirred at 80℃. After 12 h, the reaction was quenched by NH_4_Cl(aq) and extracted with methylene chloride. The combined organic layer was dried over anhydrous MgSO_4_ and evaporated under a vacuum. The mixture was purified by silica gel column chromatography with hexane as eluent. The brown solid of **6** was collected in 73% yield (2.27 g, 4.92 mmol). ^1^H NMR (400 MHz, 298 K, CDCl_3_) *δ* 8.55 (d, 2H, *J* = 2.1 Hz), 7.68 (d, 1H, *J* = 2.1 Hz), 7.65~7.52 (m, 5H), 7.46~7.39 (m, 3H) , 1.46 (s, 9H), 1.28 (s, 9H), 0.43 (s, 9H). ^13^C NMR (100 MHz, 298 K, CDCl_3_) *δ* 148.7, 147.6, 138.9, 138.1, 132.7, 131.4, 131.3, 129.6, 128.7, 128.4, 127.7, 127.1, 126.8, 126.0, 125.1, 121.7, 121.5, 116.5, 106.0, 102.5, 35.4, 35.2, 31.1, 31.0, 0.6.

*9,9'-bianthracene* (**7**)

A mixture of anthraquinone (2.0 g, 9.51mmol), zinc (4.4 g, 67.2 mmol), and HOAc (48 mL) wasplaced in a three-necked flask and added HCl (12mL) dropwise under N_2_. The mixture was stirred at 80℃. After 8 h, the reaction was quenched by NaHCO_3_(aq) and extracted with methylene chloride. The combined organic layer was dried over anhydrous MgSO_4_ and evaporated under a vacuum. The mixture was purified by silica gel column chromatography with hexane as eluent. The white solid of **7** was collected in 70% yield (2.35 g, 6.65 mmol). ^1^H NMR (400 MHz, 298 K, CDCl_3_) *δ* 8.68 (s, 2H), 8.15 (d, 4H, *J* = 8.8 Hz), 7.46~7.42 (m, 4H), 7.16~7.07 (m, 8H). ^13^C NMR (100 MHz, 298 K, CDCl_3_) *δ* 133.1, 131.6, 131.5, 128.5, 127.2, 126.8, 125.8, 125.3.

*10,10'-dibromo-9,9'-bianthracene* (**8**)

A mixture of **7** (1.2 g, 2.34 mmol), and Br_2_ (0.39 mL, 4.68 mmol) was placed in a three-necked flask containing methylene chloride (30 mL) under N_2_. The mixture was stirred at room temperature. After 4 h, the reaction was quenched by NaHCO_3_(aq) and extracted with methylene chloride. The combined organic layer was dried over anhydrous MgSO_4_ and evaporated under a vacuum. The mixture was purified by silica gel column chromatography with hexane as eluent. The yellow solid of **8** was collected in 85% yield (1.02 g, 1.99 mmol). ^1^H NMR (400 MHz, 298 K, CDCl_3_) *δ* 8.71 (d, 4H, *J* = 8.8 Hz), 7.60~7.56 (m, 4H), 7.20~7.18 (m, 4H), 7.16~7.07 (m, 4H). ^13^C NMR (100 MHz, 298 K, CDCl_3_) *δ* 133.2, 132.2, 130.4, 128.1, 127.2, 127.1, 126.3, 123.9.

*9,10-bis((2,6-di-tert-butyl-10-phenylanthracen-9-yl)ethynyl)anthracene* (**ADF1**)

A mixture of **6** (2 g, 4.32 mmol), and K_2_CO_3_ (2.99 mL, 21.6 mmol) was placed in a three-necked flask containing MeOH (15 mL)/THF (15 mL) under N_2_. The resulted mixture was stirred at room temperature. After 4 h, the reaction was extracted with EA. The combined organic layer was dried over anhydrous MgSO_4_ and evaporated under a vacuum. The crude product was mixed with 9,10-dibromoanthracene (440 mg, 1.31mmol), CuI(7.4 mg, 0.04 mmol), and Pd(PPh_3_)_4_(75 mg, 0.07 mmol) in a three-necked flask containing *i*-Pr_2_NH(5 mL)/toluene (15 mL) under N_2_. The resulted mixture was stirred at 80℃. After 12 h, the reaction was quenched by NH_4_Cl(aq) and extracted with methylene chloride. The combined organic layer was dried over anhydrous MgSO_4_ and evaporated under a vacuum. The mixture was purified by silica gel column chromatography with hexane as eluent. The red solid of **ADF1** was collected in 76% yield (3.10 g, 3.28 mmol). ^1^H NMR (400 MHz, 298 K, CDCl_3_) *δ* 9.19 (d, 2H, *J* = 3.6 Hz), 9.18 (s, 2H), 9.02 (d, 4H, *J* = 8.8 Hz), 7.84 (d, 2H, *J* = 2 Hz), 7.82~7.79 (m, 4H), 7.72 (d, 2H, *J* = 9.2 Hz), 7.66~7.55 (m, 10H), 7.51~7.50 (m, 4H), 1.55 (s, 18H), 1.35 (s, 18H). ^13^C NMR (100 MHz, 298 K, CDCl_3_) *δ* 149.1, 147.7, 138.7, 138.5, 132.5, 131.4, 131.2, 129.8, 128.8, 127.8, 127.6, 127.3, 127.1, 126.7, 126.2, 125.1, 121.9, 121.5, 119.3, 116.7, 100.5, 97.98, 35.38, 35.1, 31.1, 30.9. HRMS (m/z, FAB) [M]^+^ calcd for C_74_H_66_: 954.5165, found 954.5160.

*1,2-bis(2,6-di-tert-butyl-10-phenylanthracen-9-yl)ethyne* (**ADF2**)

The **ADF2** compound was synthesized through the same procedure used for **ADF1**. A orange solid of **ADF2** was obtained with a yield of 70% ^1^H NMR (400 MHz, 298 K, CDCl_3_) *δ* 9.11 (d, 2H, *J* = 9.2 Hz), 9.03 (d, 2H, *J* = 1.6 Hz), 7.74 (dd, 2H, *J* = 7.2 Hz), 7.71 (d, 2H, *J* = 9.2 Hz), 7.65~7.49 (m, 14H), 1.54 (s, 18H), 1.34 (s, 18H). ^13^C NMR (100 MHz, 298 K, CDCl_3_) *δ* 148.7, 147.6, 138.8, 137.8, 132.3, 131.2, 129.7, 128.8, 128.3, 127.5, 127.2, 127.0, 125.8, 125.0, 121.7, 121.6, 117.4, 98.3. HRMS (m/z, FAB) [M]^+^ calcd for C_58_H_58_: 754.4539, found 754.4540.

*10,10'-diphenyl-9,9'-bianthracene* (**ADF3**)

The **ADF3** compound was synthesized through the same procedure used for **5**. A yellow solid of **ADF3** was obtained with a yield of 76% ^1^H NMR (400 MHz, 298 K, CDCl_3_) *δ* 7.82 (d, 2H, *J* = 8.8 Hz), 7.67~7.60 (m, 5H), 7.35~7.31 (m, 2H), 7.24 (d, 2H, *J* = 7.4 Hz), 7.18~7.14 (m, 2H). ^13^C NMR (100 MHz, 298 K, CDCl_3_) *δ* 139.7, 137.9, 133.4, 131.5,131.4, 130.1, 128.5, 127.6, 127.2, 127.1, 125.5, 125.2. HRMS (m/z, FAB) [M]^+^ calcd for C_40_H_26_: 506.2035, found 506.2033.

*2,7-dibromo-9,9-dihexyl-9H-fluorene* (**9**)

2,7-dibromo-9*H*-fluorene (2.6 g, 8.02 mmol) was dissolved in dry THF (10 mL) and the mixture was cooling to 0 ℃. KO^t^Bu (2 g, 17.8 mmol) was dissolved in THF (10 mL) was added slowly to the solution and the reaction mixture was stirred for 1 h and then returned to r.t., 1-bromohexane (4.4 mL, 31.4 mmol) was added into the reaction mixture and stirred overnight, then quenched with water and extracted with ethyl acetate. The organic layer was dried over MgSO_4,_ concentrated by rotary evaporation. The residue was purified by column chromatography on silica gel with hexanes as the eluent. Compound 9 was obtained as a white solid (81% yield). ^1^H NMR (300 MHz, CDCl_3_): δ = 7.53 (sd, *J* = 8.7 Hz, 2H), 7.43 (dd, *J*_1_ = 1.5 Hz, *J*_2_ = 1.3 Hz, 4H), 1.93-1.88 (m, 4H), 1.15-1.03 (m, 12H), 0.80-0.75 (t, *J* = 6.7 Hz, 6H), 0.57 (br s, 4H).

*7-bromo-9,9-dihexyl-N,N-bis(4-(hexyloxy)phenyl)-9H-fluoren-2-amine* (**10**)

In a nitrogen atmosphere, a mixture of 2,7-dibromo-9,9-dihexyl-9*H*-fluorene (2 g, 4.06 mmol), bis(4-(hexyloxy)phenyl)amine (500 mg, 1.35 mmol), Pd(OAc)_2_ (6 mg, 0.027 mmol), dppf (37.5 mg, 0.068 mmol) and Na*^t^*OBu (195 mg, 2.03 mmol) in dry toluene was refluxed overnight and monitored through TLC. On completion, the reaction mixture was cooled to room temperature, water was added to the reaction and then extracted with CH_2_Cl_2_. The combined organic phase was dried over MgSO_4_ and concentrated by rotary evaporation. The residue was purified by column chromatography on silica gel with hexanes: CH_2_Cl_2_ (2:1 by vol.) as the eluent. Compound 10 was obtained as a light yellow solid (79% yield). ^1^H NMR (300 MHz, CDCl_3_): δ = 7.45-7.38 (m, 4H), 7.06 (d, *J* = 8.9 Hz, 4H), 6.96 (sd, J = 1.39 Hz, 1H), 6.86 (dd, *J*_1_ = 8.3 Hz, *J*_2_ = 1.7 Hz, 1H), 6.83 (d, *J* = 8.8 Hz, 4H), 3.94 (t, *J* = 6.5 Hz, 4H), 1.83-1.79 (m, 8H), 1.56-1.13 (m, 12H), 1.17-1.06 (m, 12H), 0.94-0.90 (m, 6H), 0.84-0.79 (m, 6H), 0.64 (br s, 4H).

*5-(7-(bis(4-(hexyloxy)phenyl)amino)-9,9-dihexyl-9H-fluoren-2-yl)thiophene-2-carbaldehyde* (**11**)

In nitrogen atmosphere, a mixture of (7-(dihexylamino)-9,9-dihexyl-9*H*-fluoren-2-yl) boronic acid (478 mg, 0.64 mmol), 5-bromothiophene-2-carbaldehyde (122 mg, 0.64 mmol)、Pd(PPh_3_)_4_ (37 mg, 0.032 mmol) and K_2_CO_3_ (533 mg, 2 M) was dissolved in dry toluene (4 mL) and dry THF (2 mL) then refluxed overnight. After cooling down to room temperature, the reaction mixture was passed through celite and extracted with CH_2_Cl_2_ and deionized water. The organic layer was dried over anhydrous MgSO_4_ and evaporated to give the crude product. It was further purified through column chromatography by using silica gel as stationary phase and hexanes: CH_2_Cl_2_ (4:1 by vol.) as eluent to give 11 as an orange solid (69% yield). ^1^H NMR (300 MHz, CDCl_3_): δ = 9.88 (s, 1H), 7.75 (d, *J* = 3.9 Hz, 1H), 7.63-7.59 (m, 2H), 7.56 (sd, *J* = 3.8 Hz, 1H), 7.50 (d, *J* = 8.4 Hz, 1H), 7.44(d, *J* = 3.8 Hz, 1H), 6.98 (d, *J* = 8.9 Hz, 4H), 6.90(sd, *J* = 1.7 Hz, 1H), 6.88(dd, *J*_1_ = 1.8 Hz, *J*_2_ = 1.9 Hz, 1H), 6.84 (d, *J* = 8.9 Hz, 4H), 3.94 (t, *J* = 6.5 Hz, 4H), 1.90-1.74 (m, 8H), 1.49-1.34 (m, 12H), 1.16-1.06 (m, 12H), 0.94-0.89 (m, 6H), 0.82-0.77 (m, 6H), 0.68 (br s, 4H).

YI-1, YI-3 and YI-8 were synthesized via similiar procedures. Only the synthesis of YI-1 is described in detail.

(*E*)-2-(2-(5-(7-(bis(4-(hexyloxy)phenyl)amino)-9,9-dihexyl-9*H*-fluoren-2-yl)thiophen-2-yl)vinyl)-5-carboxy-1-hexyl-3,3-dimethyl-3*H*-indol-1-ium iodide (**YI-1**)

A solution of 11 (350 mg, 0.43 mmol), 5-carboxy-2,3,3-trimethyl-1-hexyl-3*H*-indolium iodide (214.7 mg, 0.52 mmol) and piperidine (10 mg, 0.12 mmol) in acetonitrile, was refluxed under nitrogen for 6 h and monitored through TLC. After cooling, the used DCM transfer to the round-bottom flask then concentrated by rotary evaporation to give the crude product. Recrystallize by hexane, diethyl ether or ethyl acetate to wash the residue, then through suction filtration and take the solid. **YI-1** was obtained as a deep blue solid (56% yield). ^1^H NMR (600 MHz, DMSO): δ = 8.82 (d, *J* = 15.6 Hz, 1H), 8.40 (s, 1H), 8.29 (sd, *J* = 3.6 Hz, 1H), 8.16 (d, *J* = 8.4 Hz, 1H), 7.97 (m, 2H), 7.83 (s, 1H), 7.79 (dd, *J* = 7.8 Hz, 2H), 7.66 (d, *J* = 8.4 Hz, 1H), 7.26 (d, *J* = 15.6 Hz, 1H), 6.98 (d, *J* = 9.0 Hz, 4H), 6.88 (s, 1H), 6.86 (d, *J* = 9.0 Hz, 4H), 6.77 (d, *J* = 9.0 Hz, 1H), 4.62 (s, 2H), 3.91 (t, *J* = 6.6 Hz, 4H), 1.91-1.79 (m, 8H), 1.69-1.63 (m, 12H), 1.44-1.32 (m, 12H), 1.30-1.00 (m, 18H), 0.87-0.59 (m, 15H); ^13^C NMR (150 MHz, DMSO): δ = 182.11, 166.49, 156.47, 155.02, 152.06, 151.12, 148.83, 147.43, 144.14, 143.58, 143.03, 140.13, 138.82, 132.31, 130.75, 130.55, 129.77, 126.52, 126.07, 125.73, 123.91, 121.31, 120.16, 119.70, 119.45, 115.33, 114.76, 114.67, 109.11, 67.58, 54.58, 51.80, 46.06, 43.73, 40.04, 30.95, 30.74, 30.69, 28.76, 28.65, 27.87, 25.81, 25.45, 25.16, 23.29, 22.16; MS (HR-FAB) calculated for C_72_H_93_N_2_O_4_S^+^: 1081.6856, found 1081.6866.

(*E*)-2-(4-(7-(bis(4-(hexyloxy)phenyl)amino)-9,9-dihexyl-9*H*-fluoren-2-yl)styryl)-5-carboxy-1-hexyl-3,3-dimethyl-3*H*-indol-1-ium iodide (**YI-3**)

**YI-3** was obtained as a green solid (31% yield). ^1^H NMR (600 MHz, CD_2_Cl_2_): δ = 8.38 (d, *J* = 15.0 Hz, 1H), 8.08 (d, *J* = 7.8 Hz, 2H), 7.95 (d, *J* = 7.8 Hz, 2H), 7.70 (sd, *J* = 5.4 Hz, 2H), 7.67 (s, 1H), 7.54 (d, *J* = 8.4 Hz, 1H), 7.48 (d, *J* = 16.2 Hz, 1H), 7.06-7.00 (m, 6H), 6.88 (d, *J* = 7.8 Hz, 1H), 6.84 (d, *J* = 9.0 Hz, 6H), 4.67 (s, 2H), 3.95 (t, *J* = 6.6 Hz, 4H), 6.78 (d, *J* = 8.2 Hz, 1H), 4.76 (s, 2H), 3.93 (t, *J* = 6.2 Hz, 4H), 1.95-1.82 (m, 8H), 1.80-1.70 (m, 12H), 1.63-1.40 (m, 12H), 1.36-1.14 (m, 18H), 0.92-0.69 (m, 15H); ^13^C NMR (150 MHz, CD_2_Cl_2_): δ = 183.39, 156.25, 155.87, 153.02, 152.78, 151.99, 149.51, 148.63, 143.62, 143.16, 141.38, 136.52, 135.36, 133.47, 132.43, 132.18, 130.50, 128.30, 127.78, 126.76, 126.62, 124.93, 121.73, 120.92, 120.46, 119.63, 119.46, 116.36, 115,58, 115.11, 111.29, 68.72, 55.57, 48.94, 45.17, 40.58, 32.00, 31.92, 31.69, 30.00, 29.72, 29.21, 28.67, 28.51, 28.02, 27.56, 26.98, 26.42, 26.11. MS (HR-FAB) calculated for C_74_H_95_N_2_O_4_^+^: 1075.7292, found 1075.7292.

(*E*)-2-(4-(bis(4-(hexyloxy)phenyl)amino)styryl)-5-carboxy-1-hexyl-3,3-dimethyl-3*H*-indol-1-ium iodide (**YI-8**)

**YI-8** was obtained as a blue solid (43% yield). ^1^H NMR (600 MHz, CD_2_Cl_2_): δ = 8.30-8.25 (m, 2H), 8.14 (d, *J* = 15.6 Hz, 1H), 7.75 (d, *J* = 8.4 Hz, 2H), 7.49 (d, *J* = 8.4 Hz, 1H), 7.17 (d, *J* = 8.4 Hz, 4H), 7.03 (d, *J* = 15.0 Hz, 1H), 6.95 (d, *J* = 8.4 Hz, 4H), 6.89 (d, *J* = 9.0 Hz, 2H), 4.43 (t, *J* = 6.6 Hz, 2H), 3.97 (t, *J* = 6.6 Hz, 4H), 1.93-1.70 (m, 14H), 1.48-1.41 (m, 8H), 1.26 (s, 8H), 0.92-0.91 (m, 9H); ^13^C NMR (150 MHz, CD_2_Cl_2_): δ = 180.79, 158.31, 155.73, 155.18, 143.76, 142.67, 137.43, 134.44, 128.50, 124.82, 117.45, 116.02, 115.82, 113.36, 105.75, 68.74, 51.82, 47.22, 44.83, 31.86, 31.62, 29.96, 29.47, 28.49, 27.75, 26.76, 25.95, 22.90, 22.69, 22.49, 22.46.; MS (HR-FAB) calculated for C_49_H_63_N_2_O_4_^+^: 743.4788, found 743.4779.

**Fig. S1.** UV-visible spectra of the Cu@PSMA NPS reacted with 1.5 M HCl as a function of the reaction time.

**
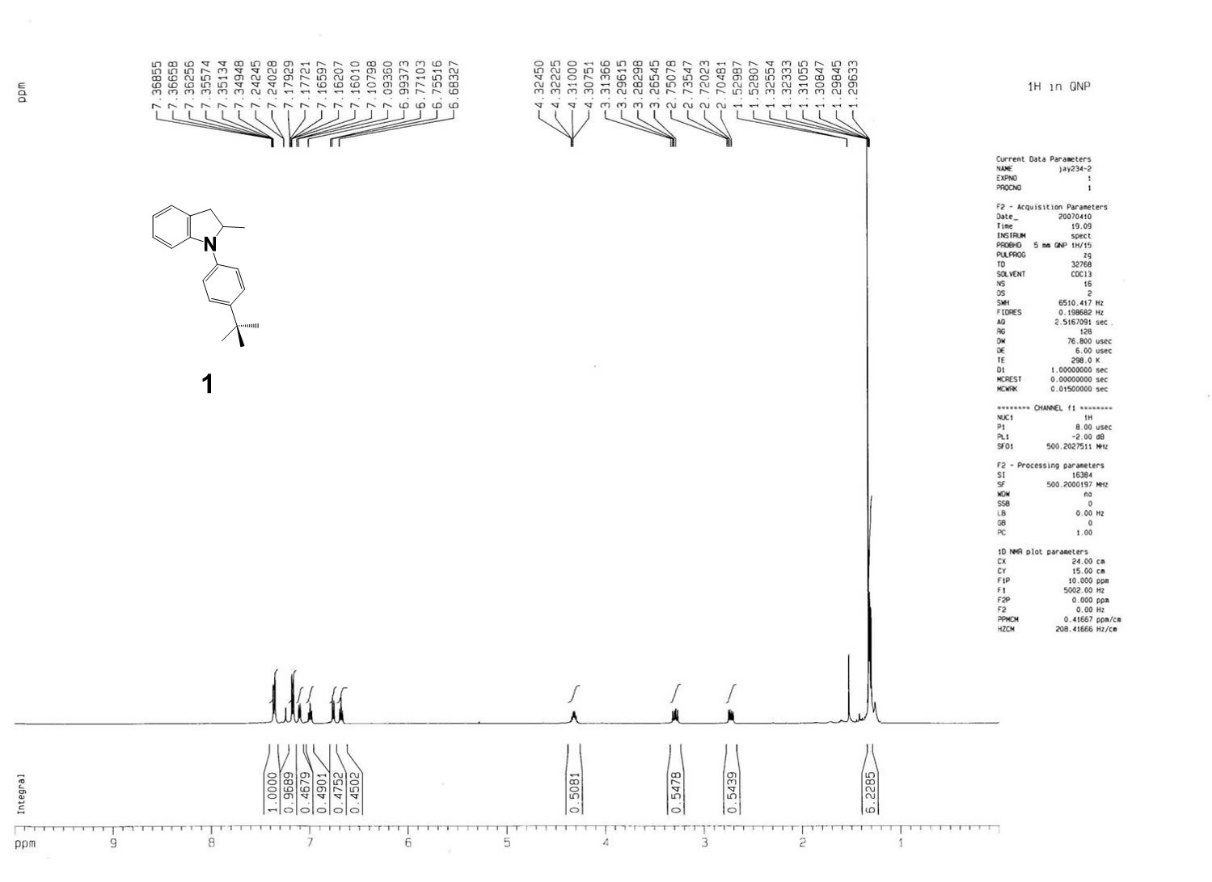
**


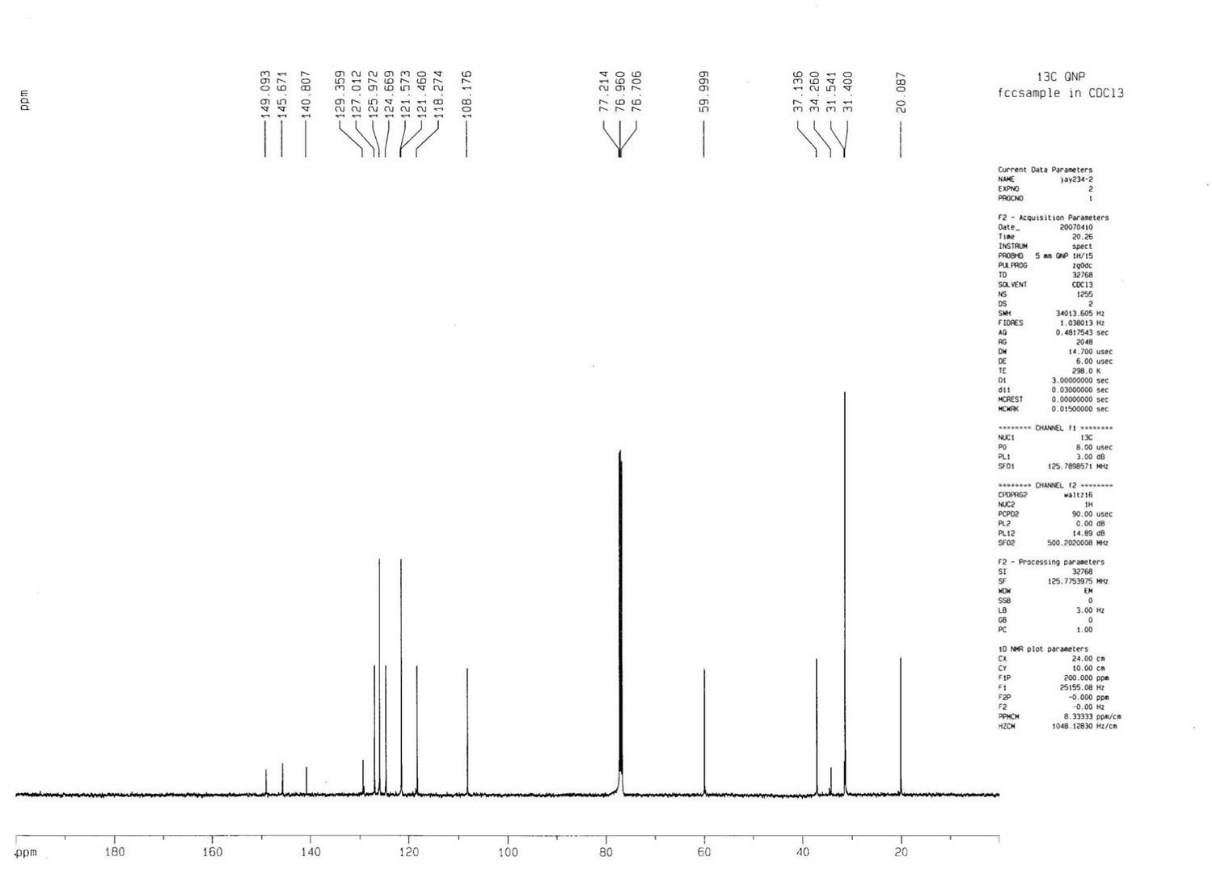


**Fig. S2.** ^1^H (top) & ^13^C NMR (bottom) spectra of **1** in CDCl_3_.


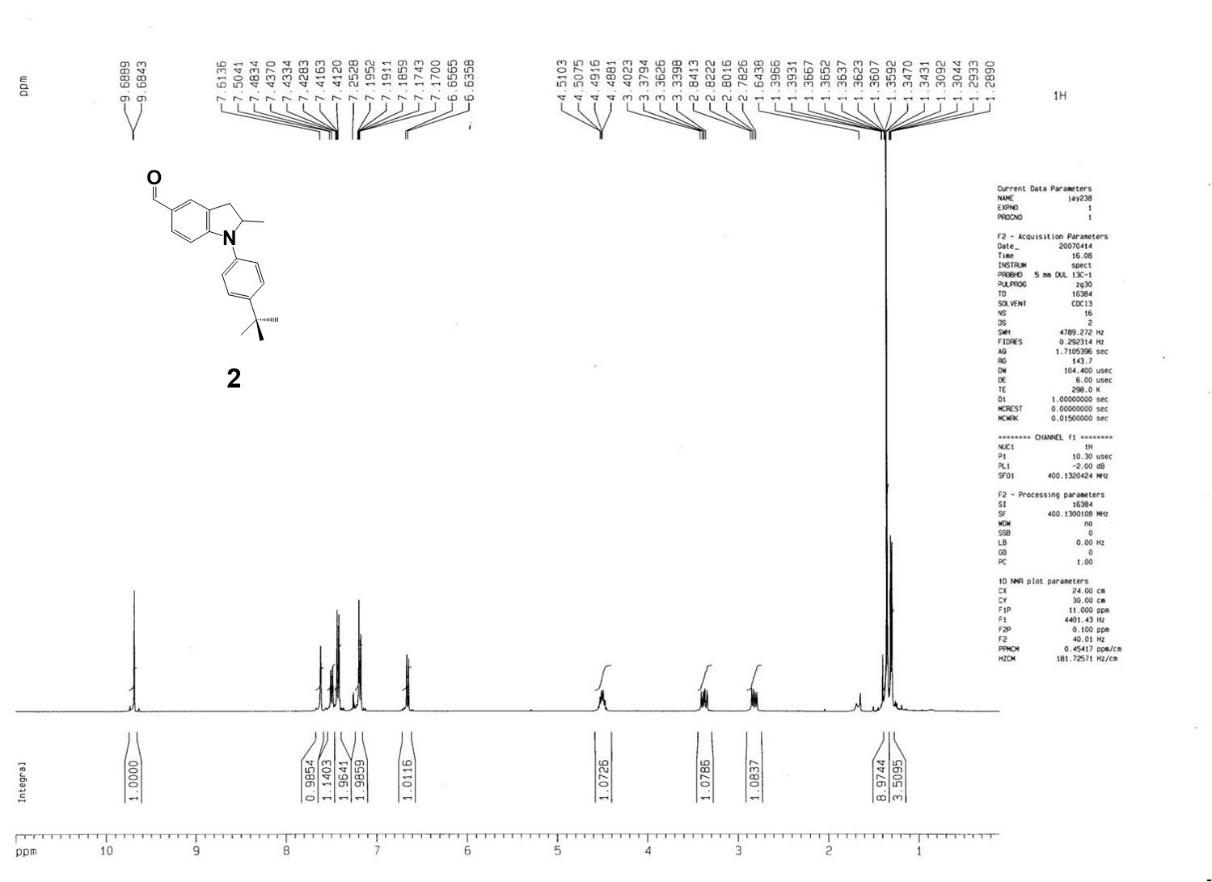


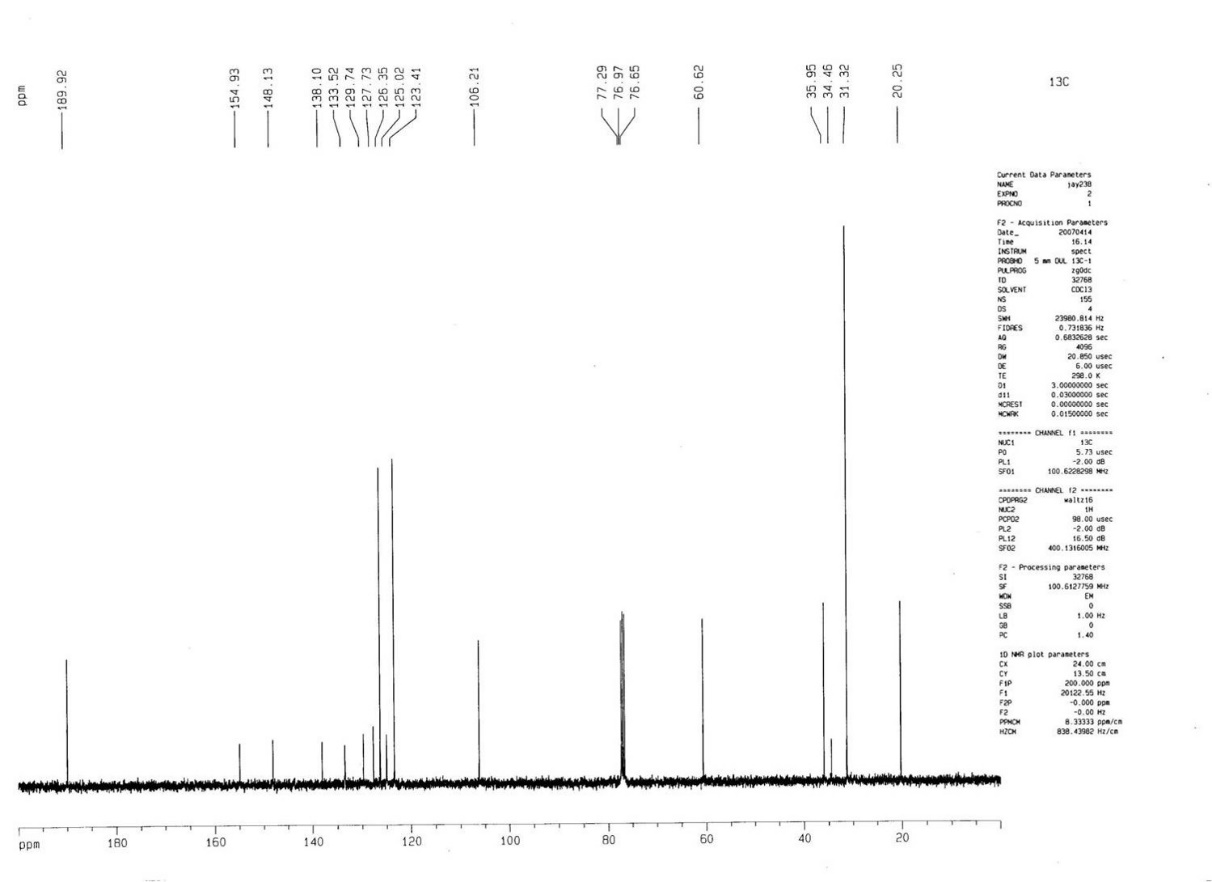


**Fig. S3.** ^1^H (top) & ^13^C NMR (bottom) spectra of **2** in CDCl_3_.

**
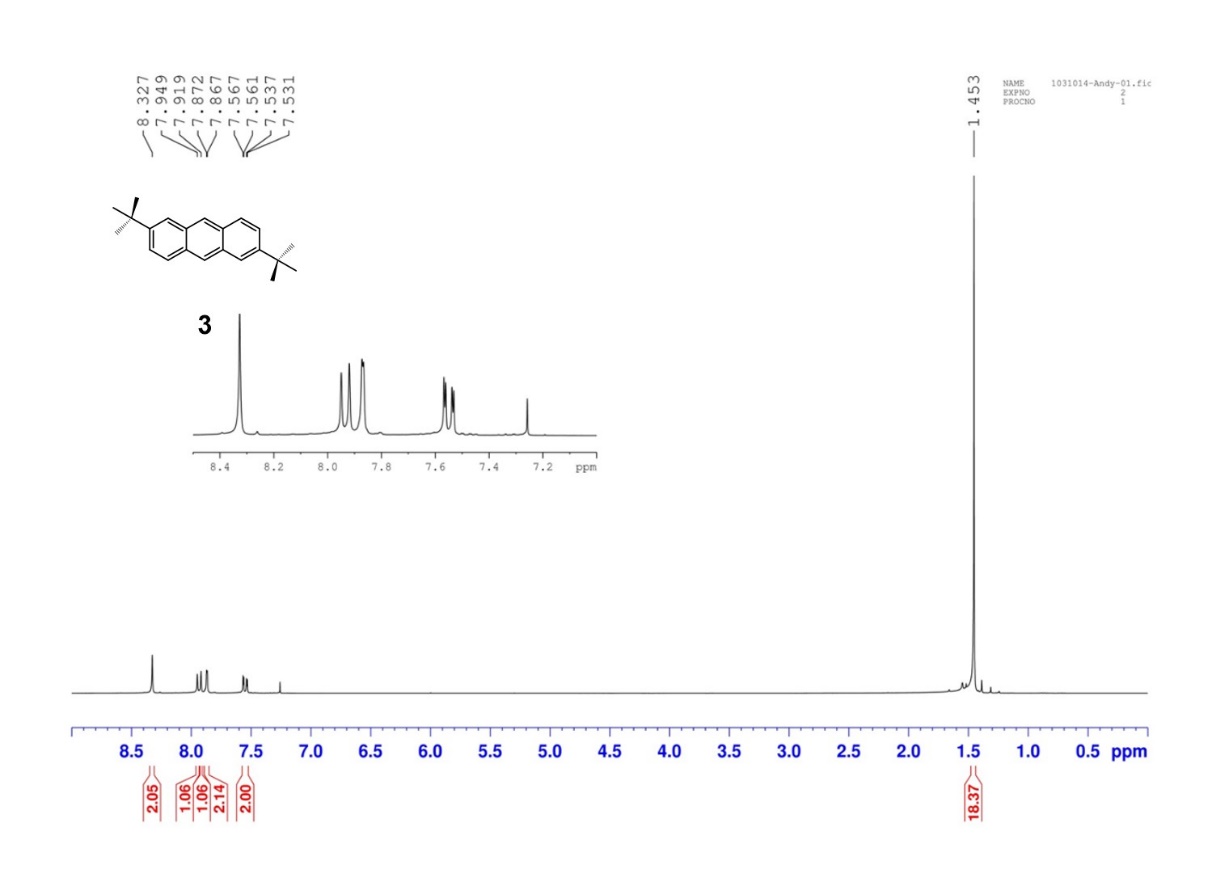
**

**
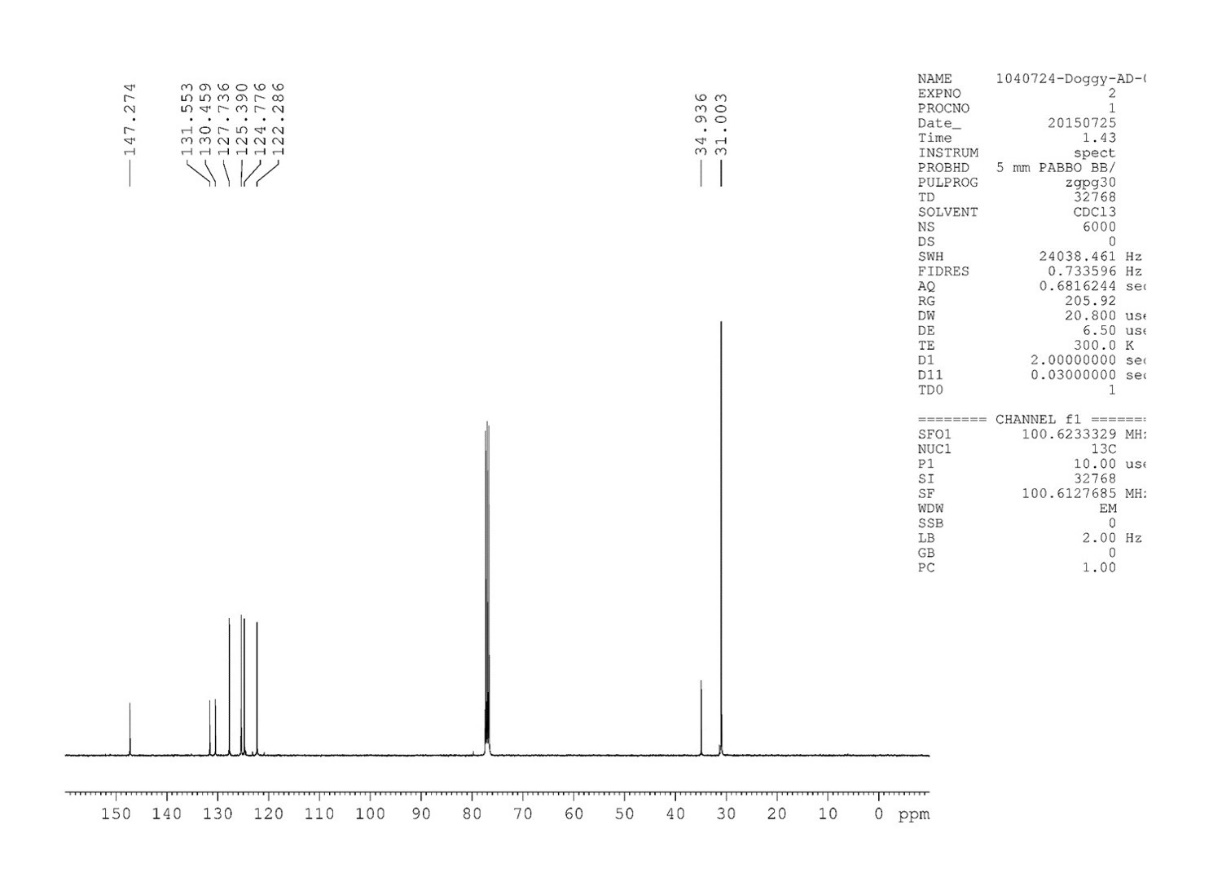
**

**Fig. S4.** ^1^H (top) & ^13^C NMR (bottom) spectra of **3** in CDCl_3_.

**
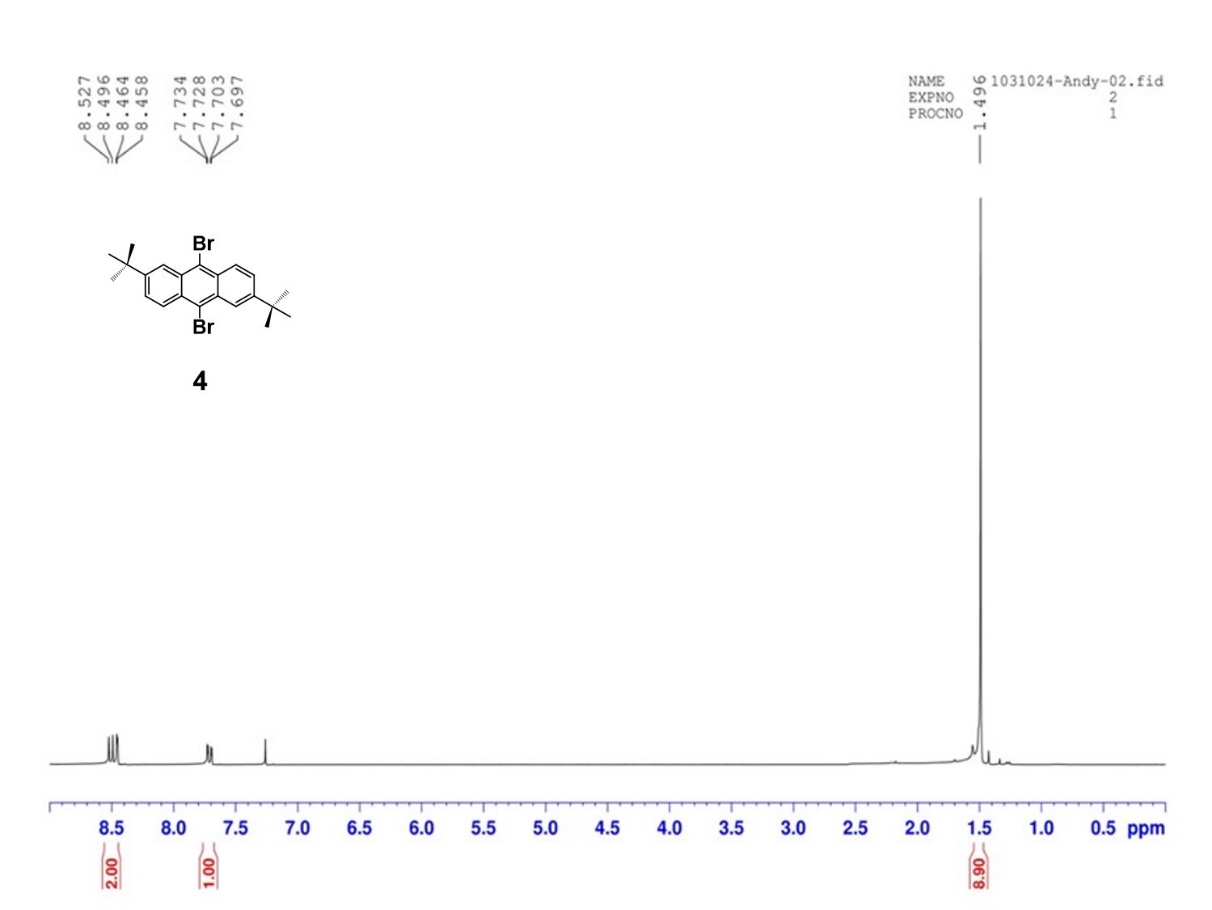
**

**
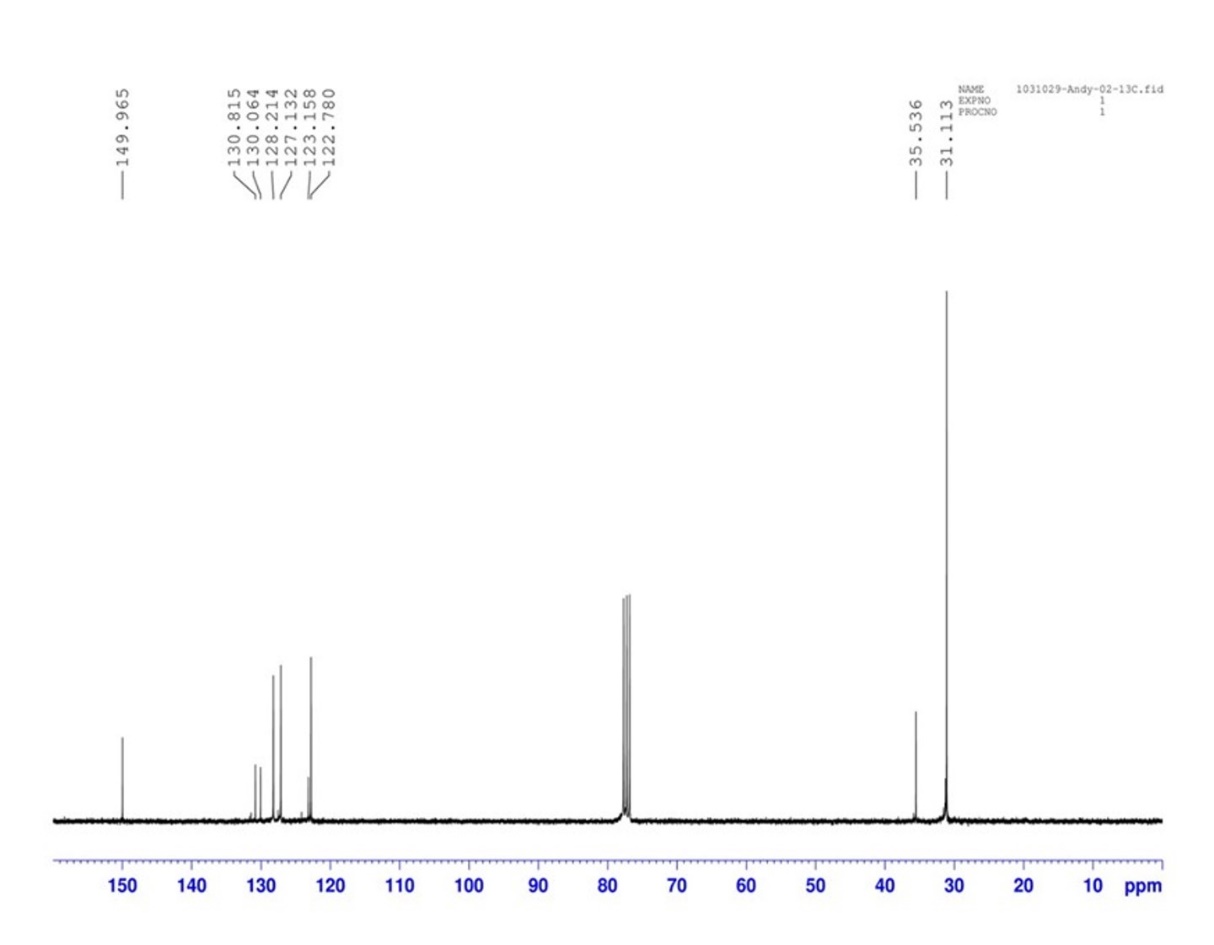
**

**Fig. S5.** ^1^H (top) & ^13^C NMR (bottom) spectra of **4** in CDCl_3_.


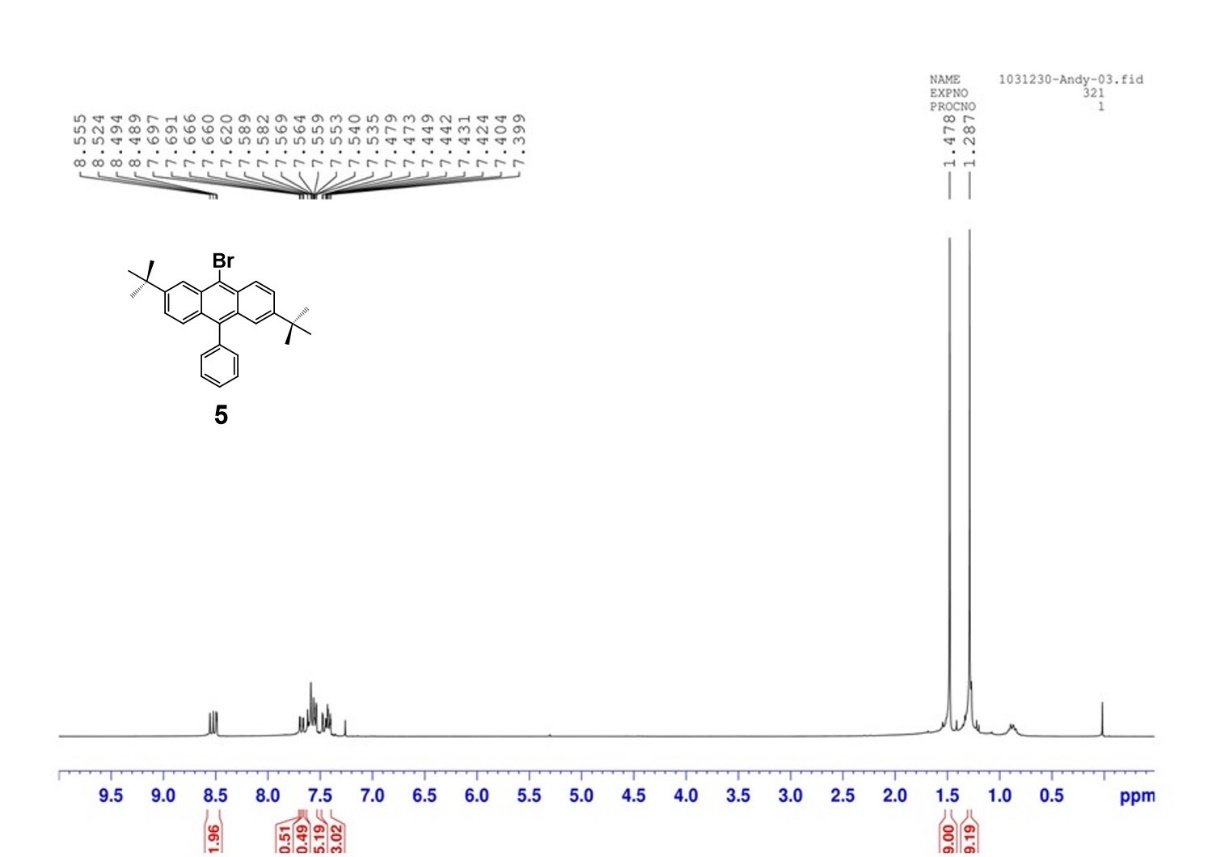


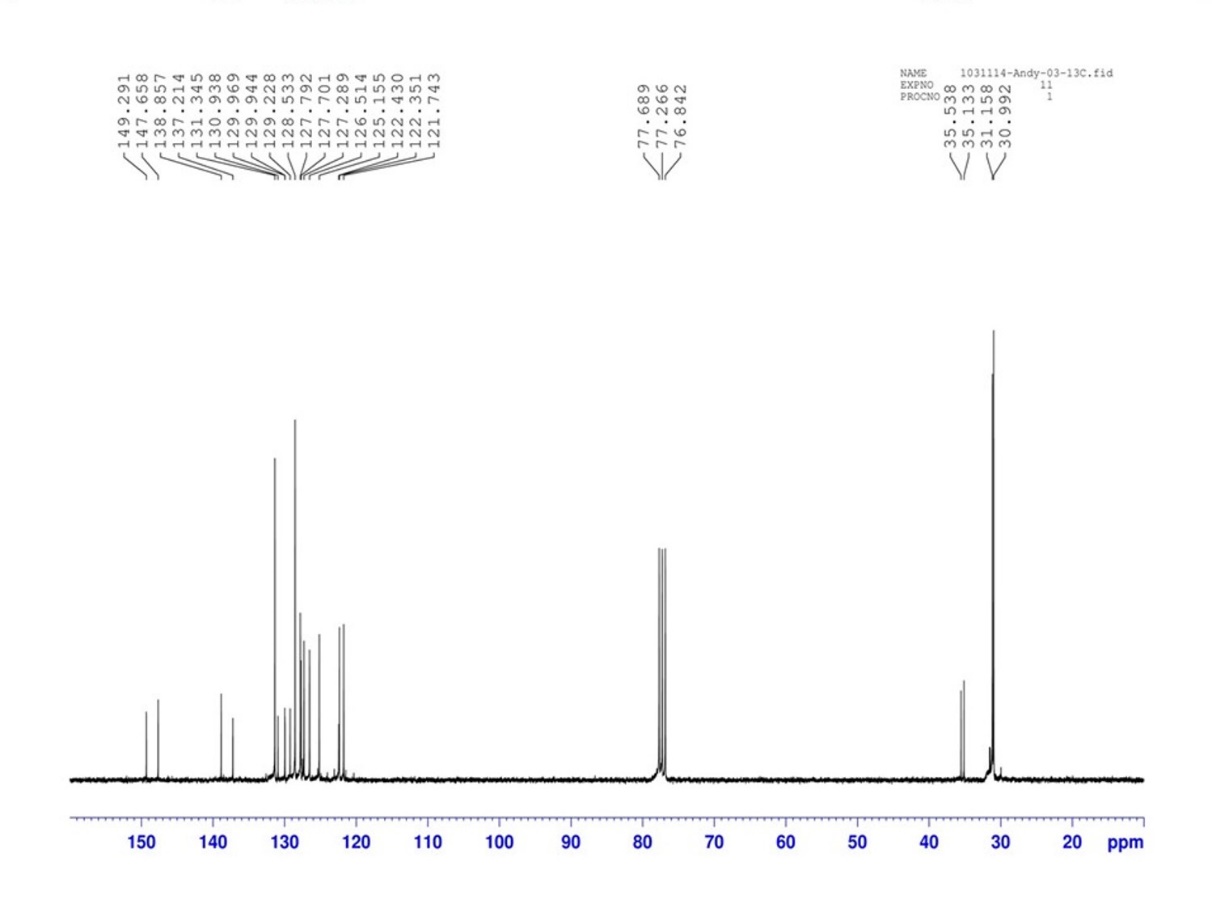


**Fig. S6.** ^1^H (top) & ^13^C NMR (bottom) spectra of **5** in CDCl_3_.


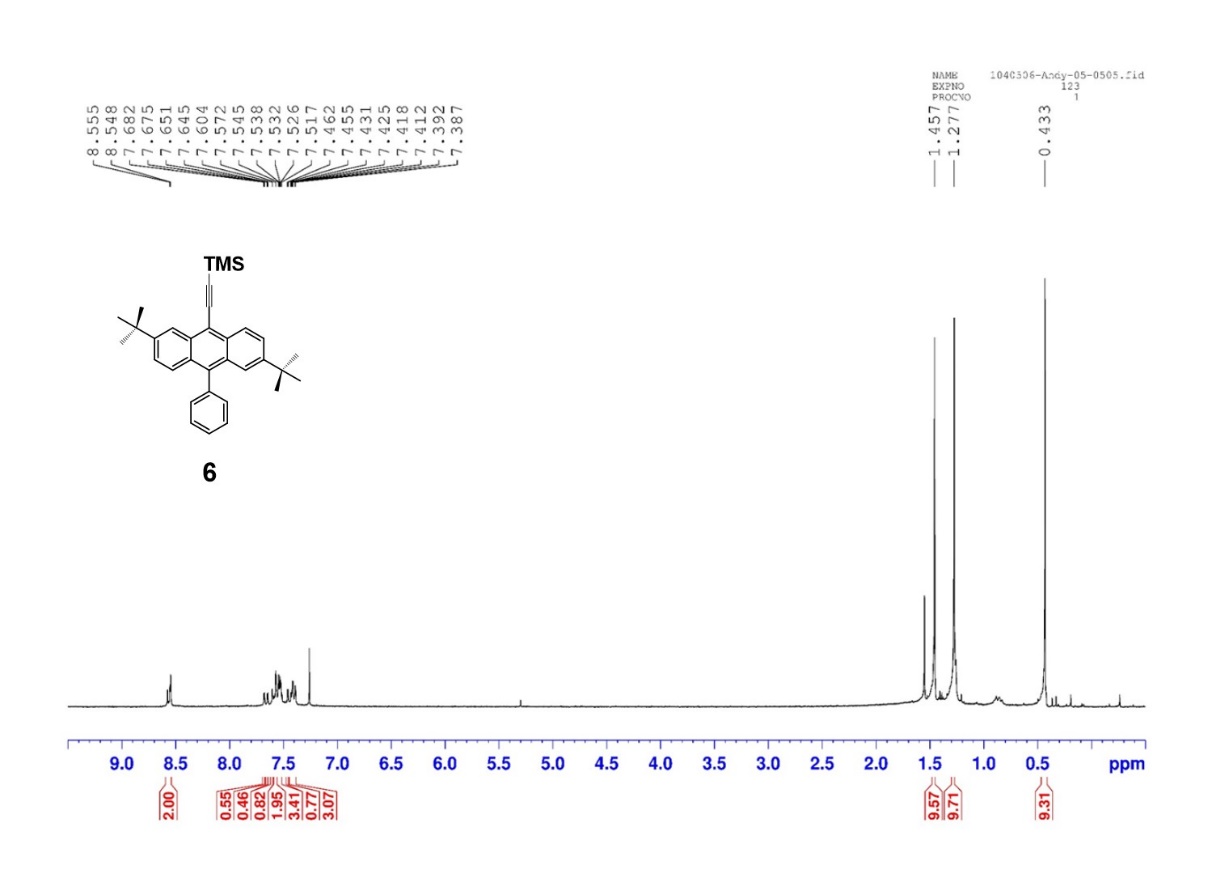


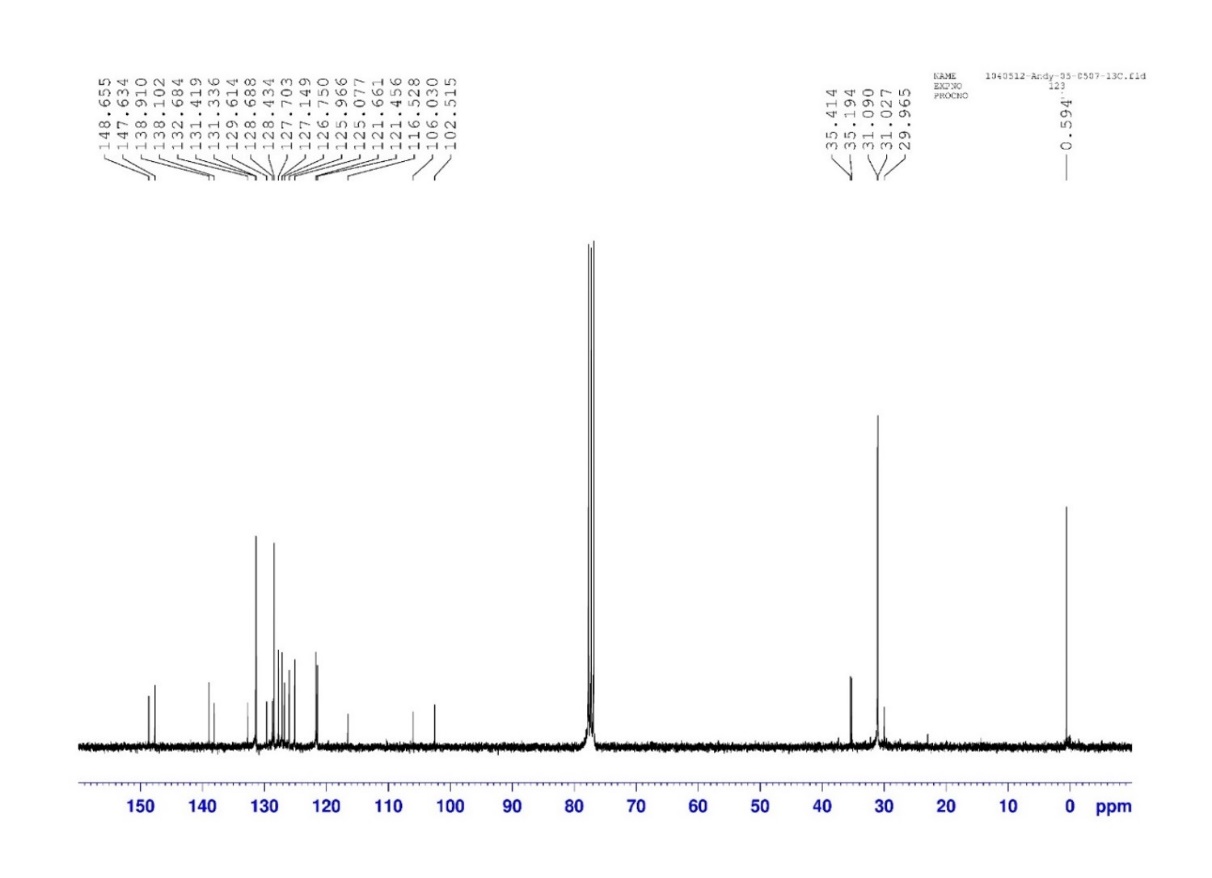


**Fig. S7.** ^1^H (top) & ^13^C NMR (bottom) spectra of **6** in CDCl_3_.


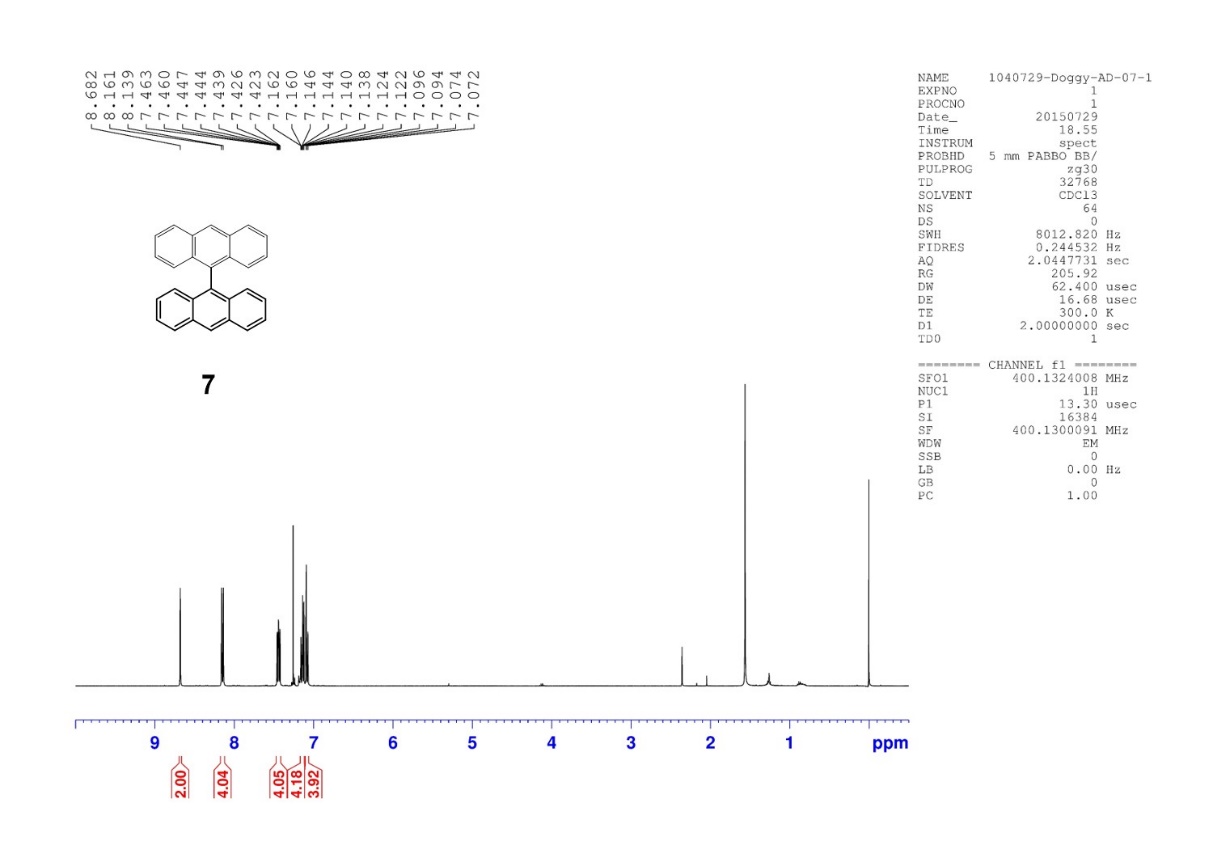


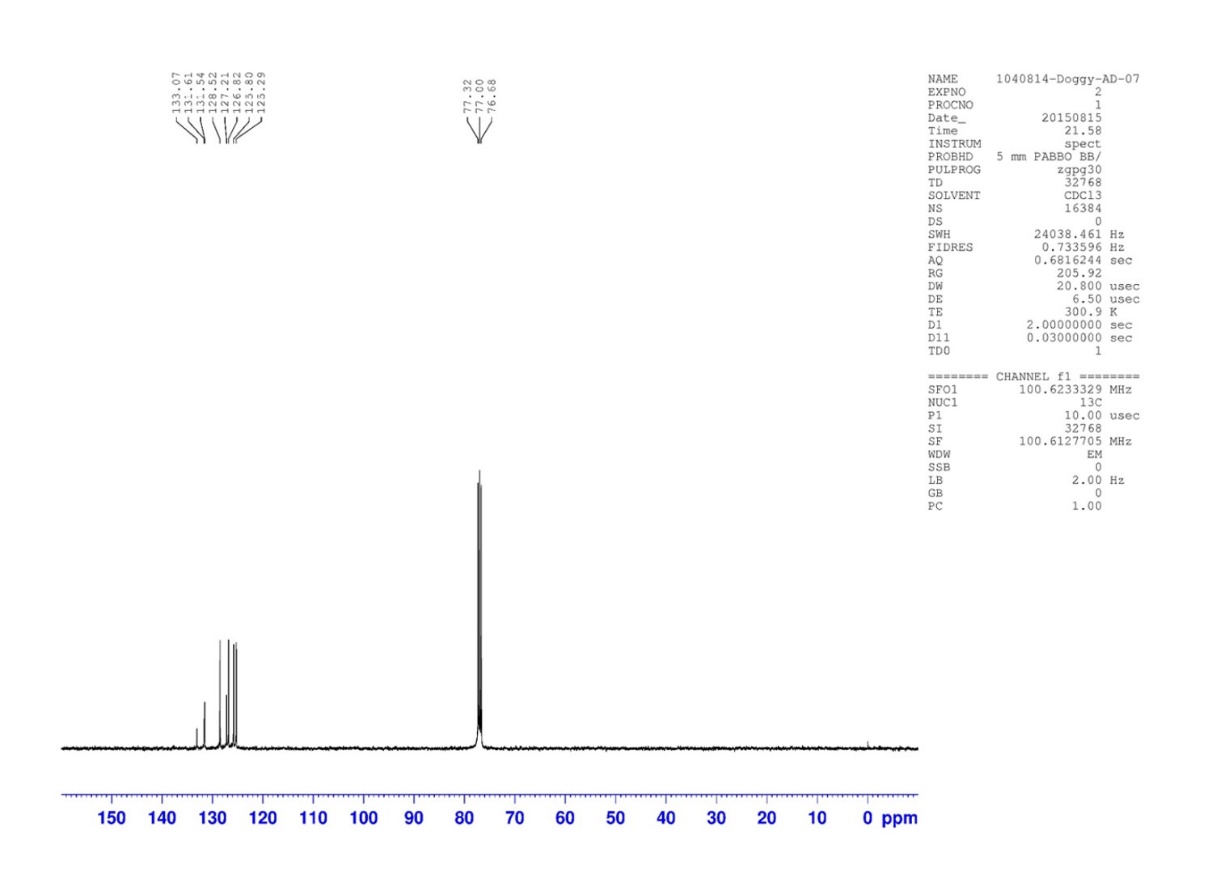


**Fig. S8.** ^1^H (top) & ^13^C NMR (bottom) spectra of **7** in CDCl_3_.


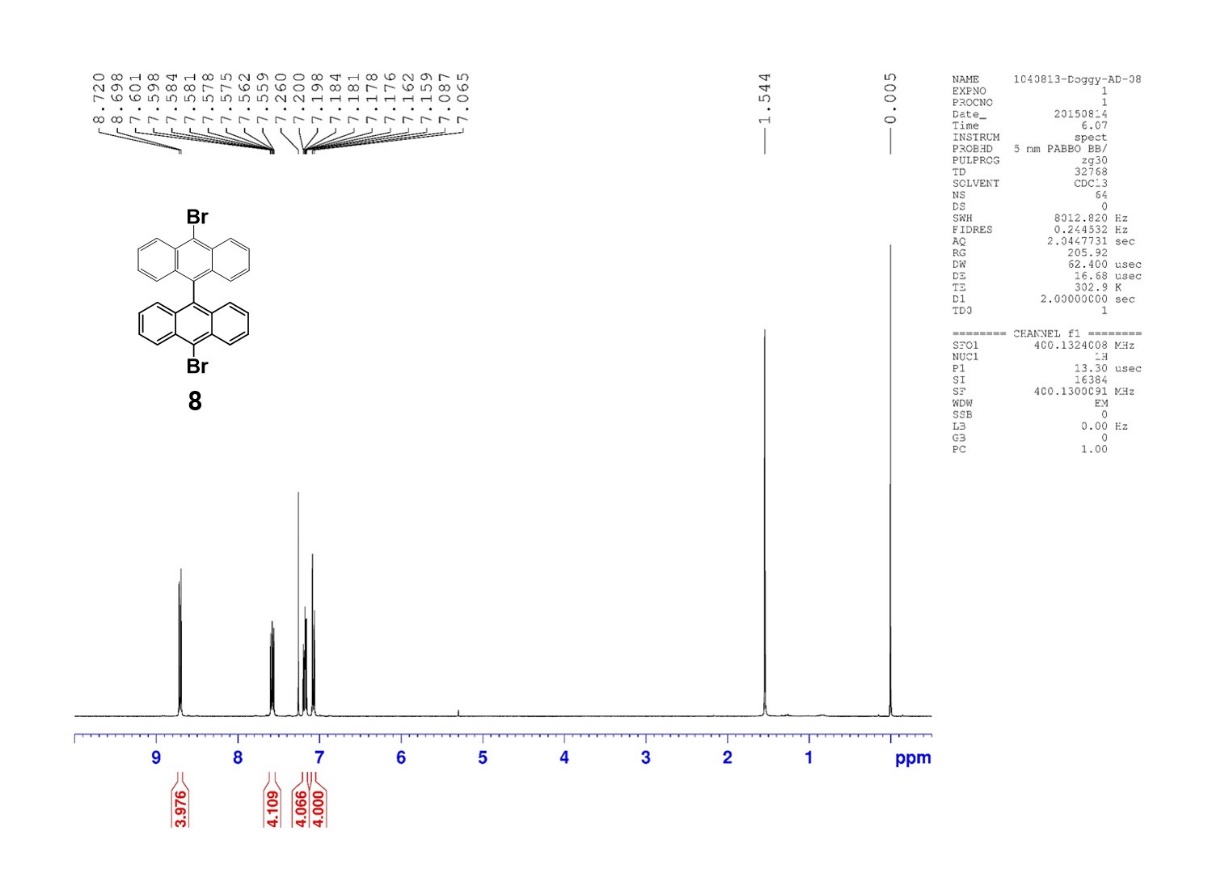


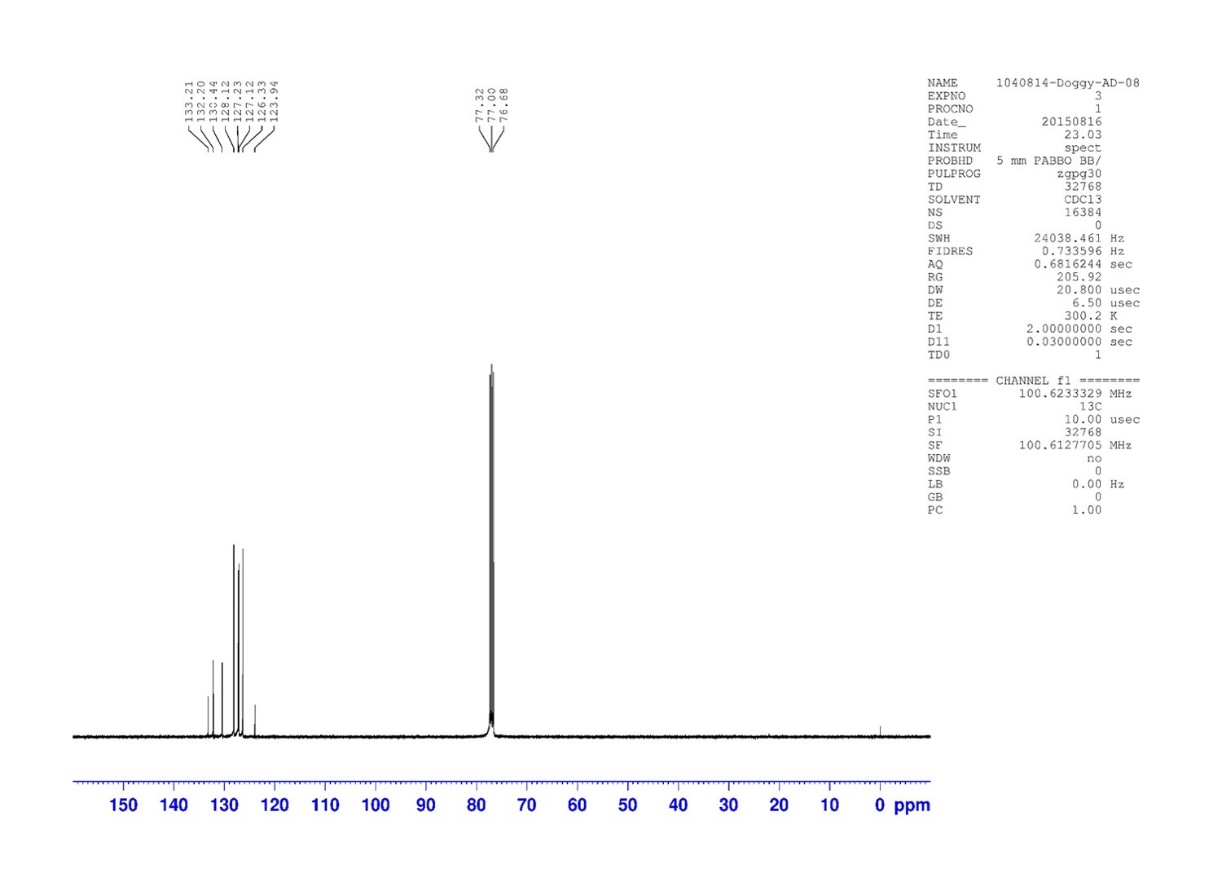


**Fig. S9.** ^1^H (top) & ^13^C NMR (bottom) spectra of **8** in CDCl_3_.


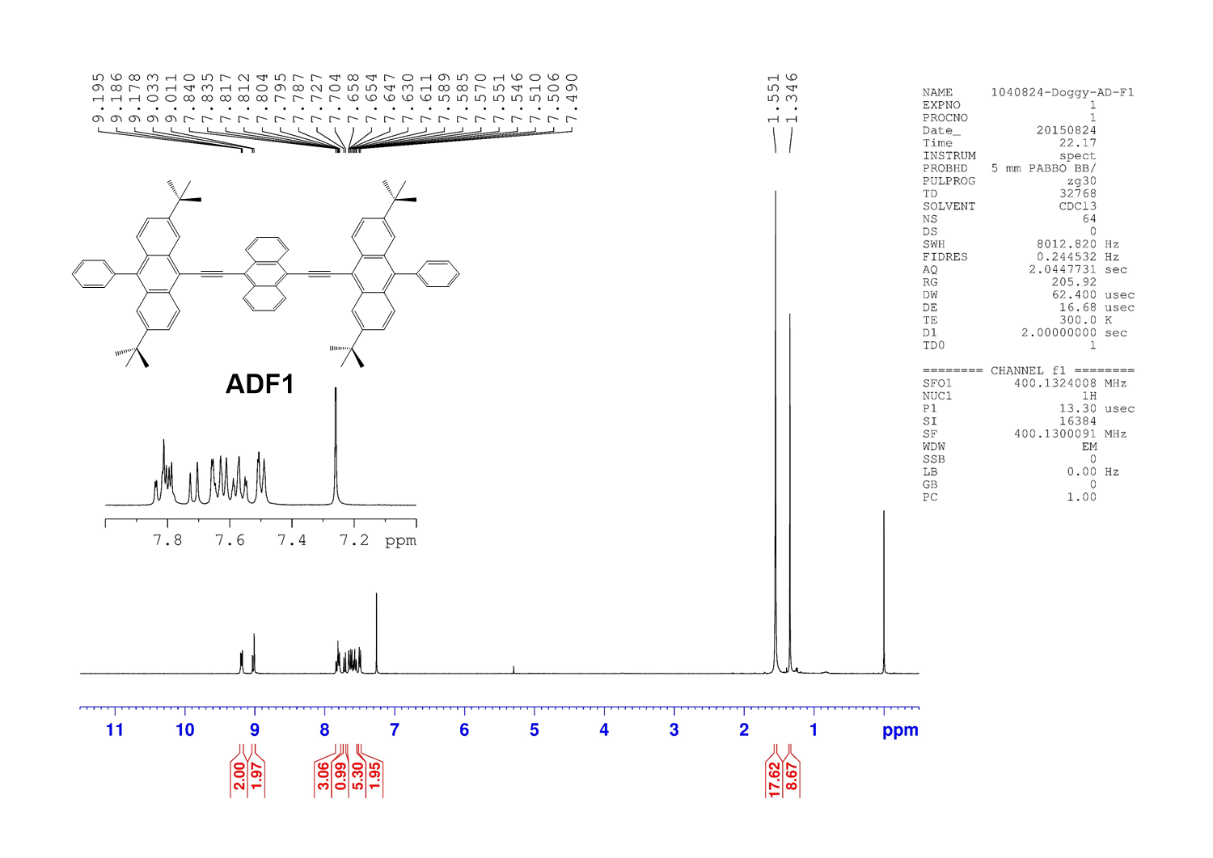


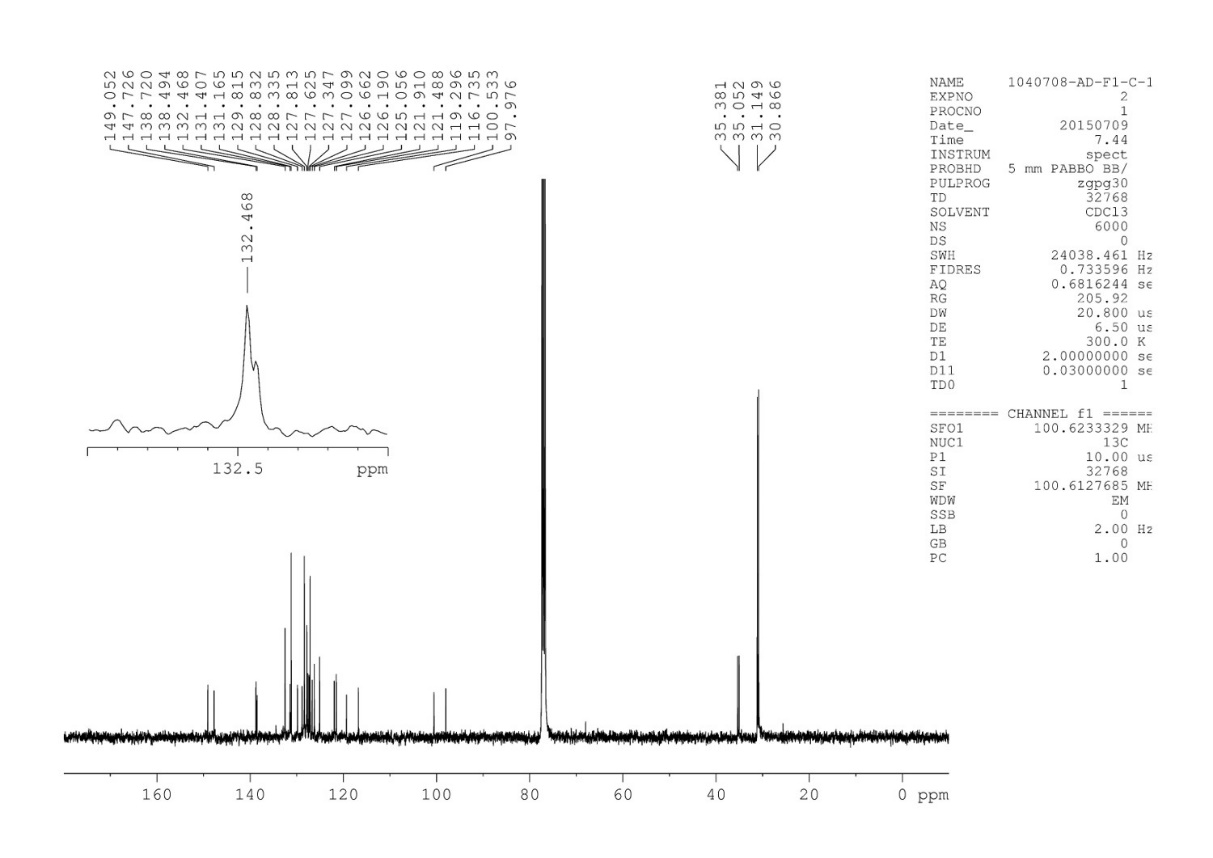


**Fig. S10.** ^1^H (top) & ^13^C NMR (bottom) spectra of **ADF1** in CDCl_3_.


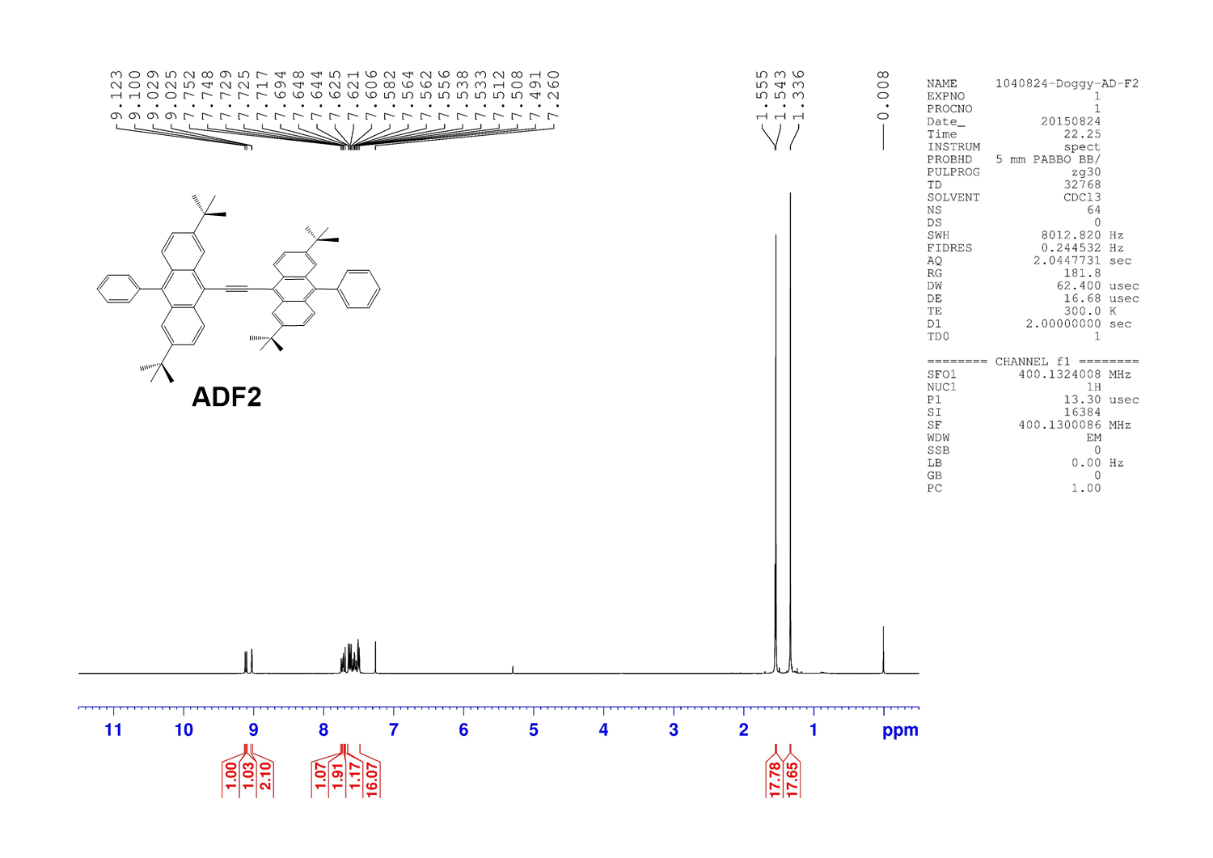


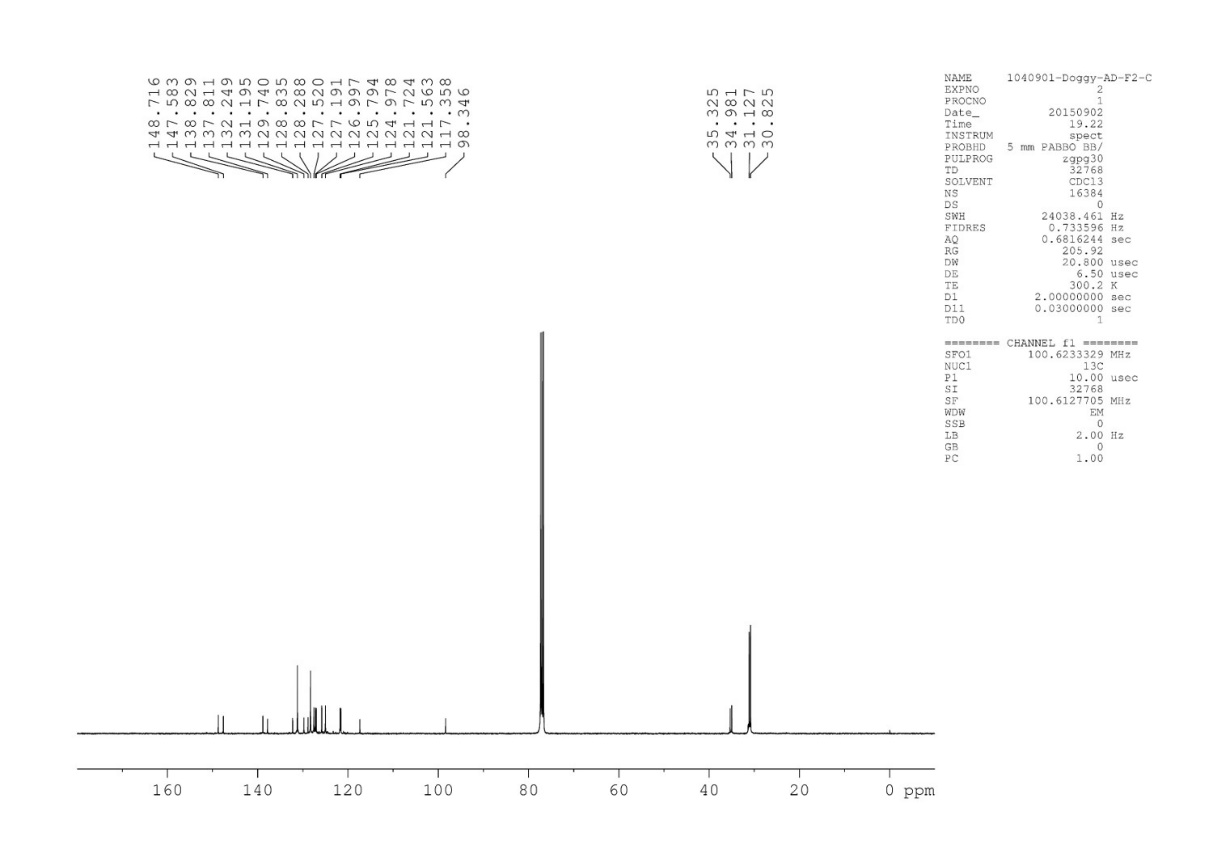


**Fig. S11.** ^1^H (top) & ^13^C NMR (bottom) spectra of **ADF2** in CDCl_3_.


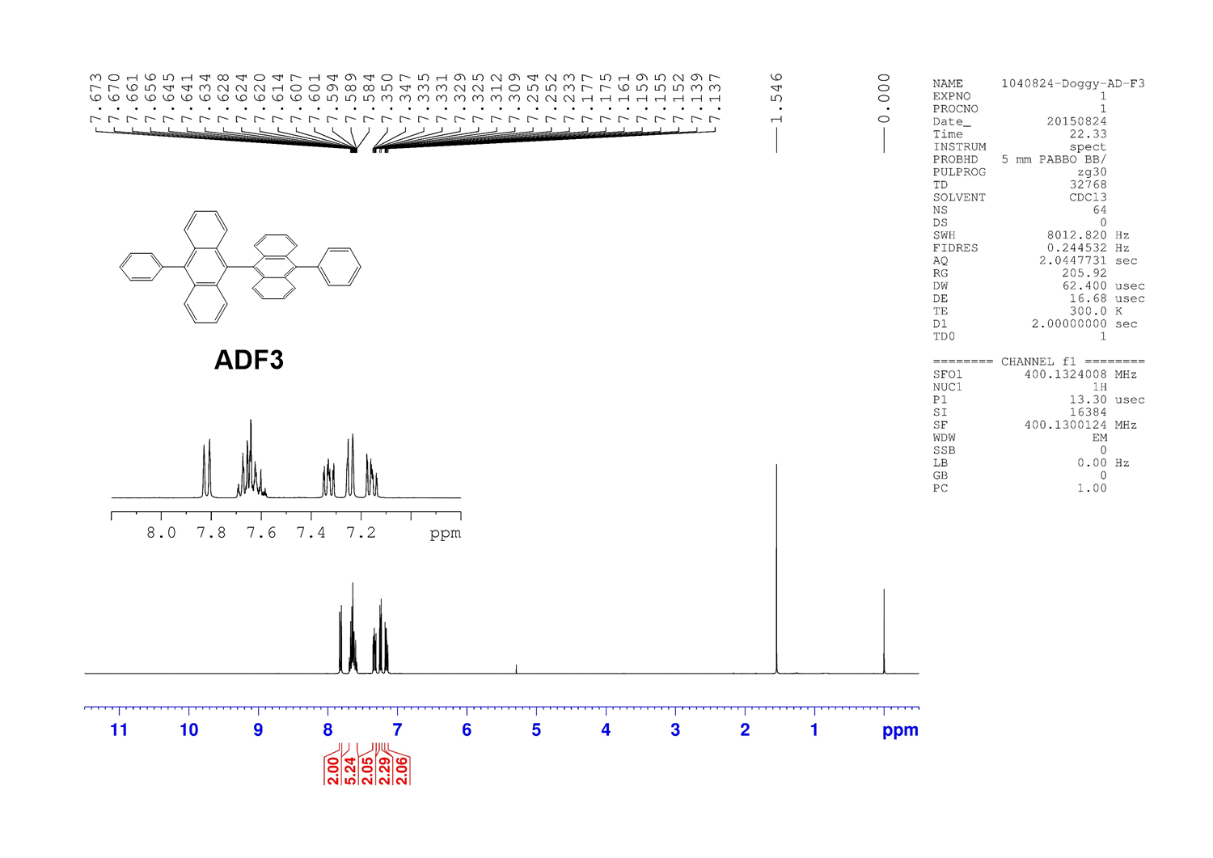


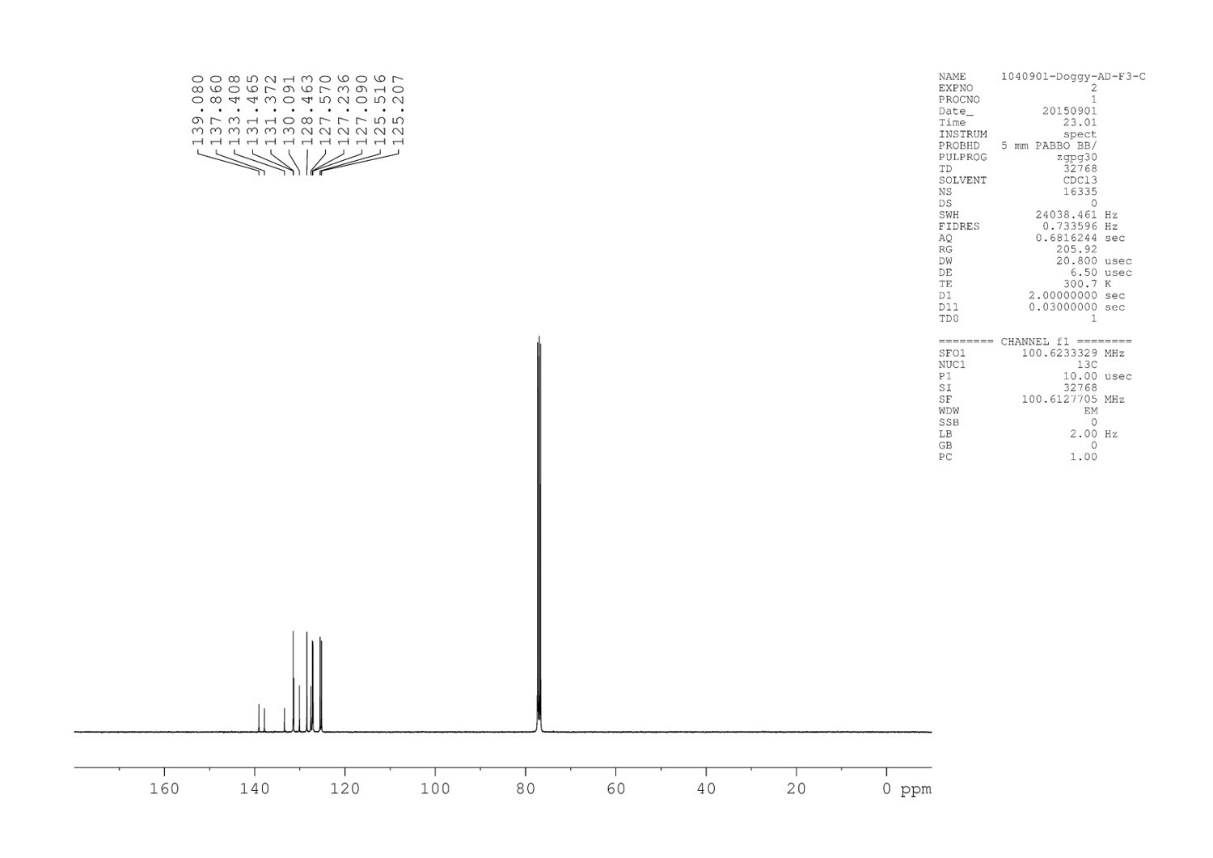


**Fig. S12.** ^1^H (top) & ^13^C NMR (bottom) spectra of **ADF3** in CDCl_3_.


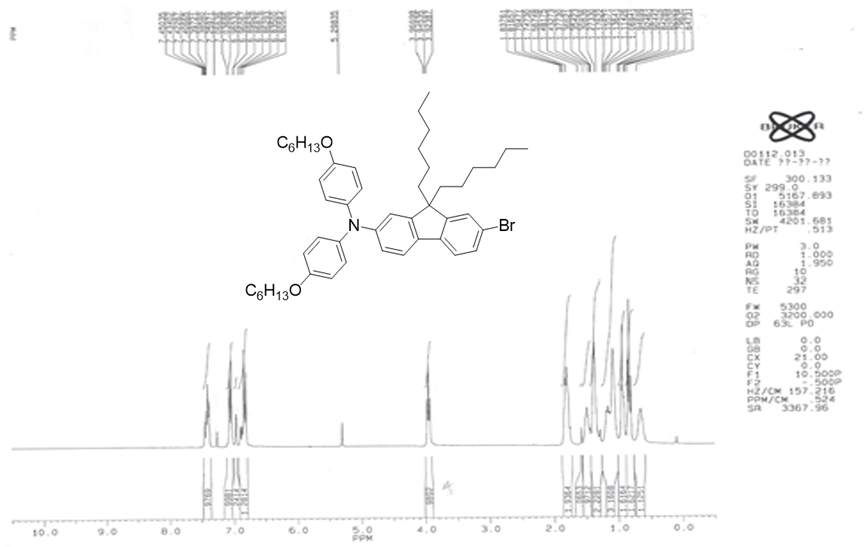


**Fig. S13.** ^1^H NMR spectra of **10** in CDCl_3_.


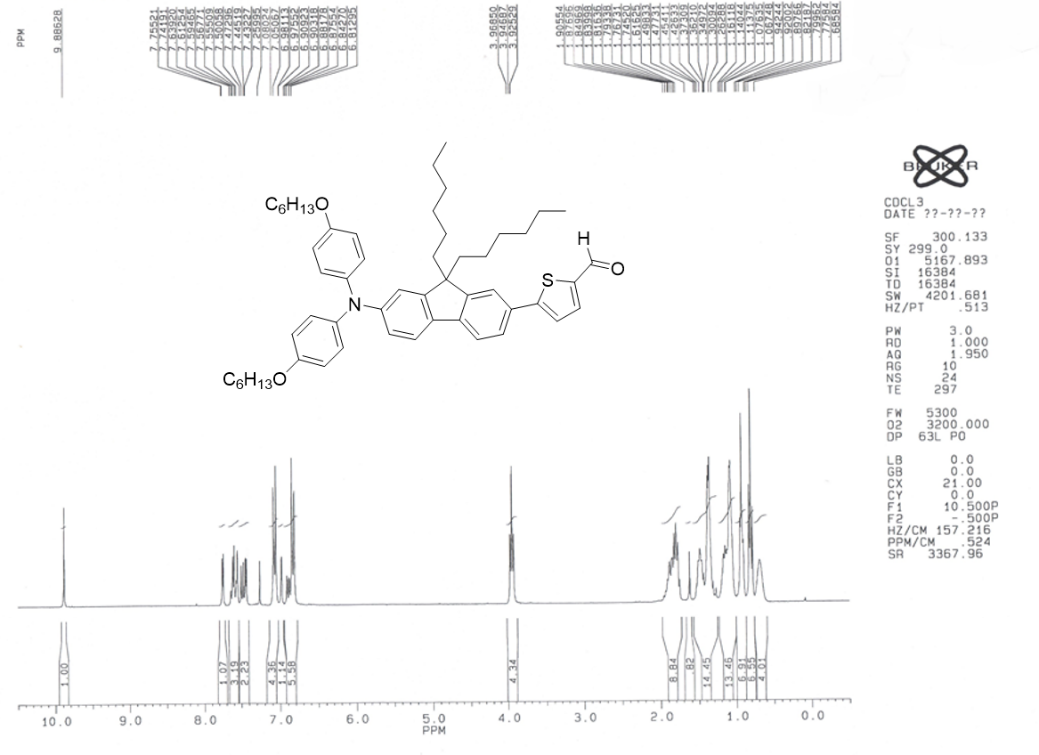


**Fig. S14.** ^1^H NMR spectra of **11** in CDCl_3_.


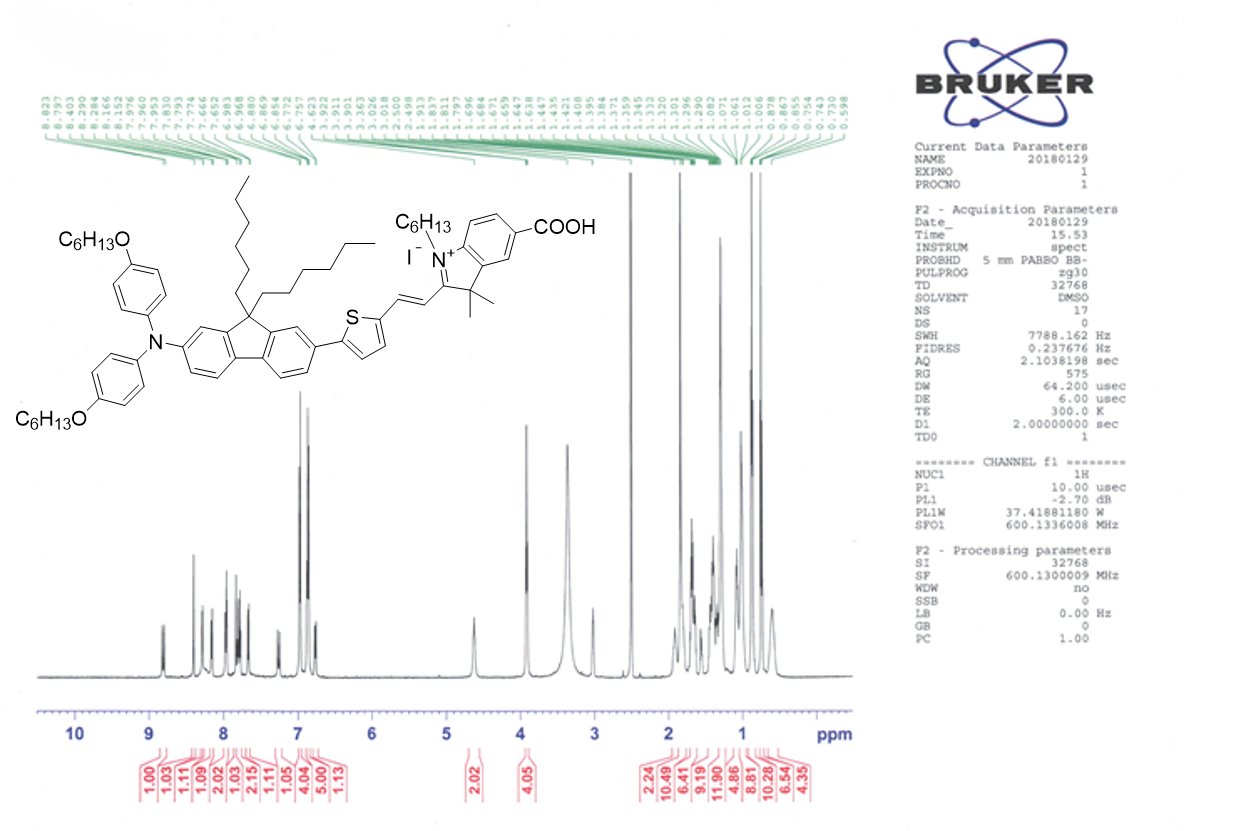


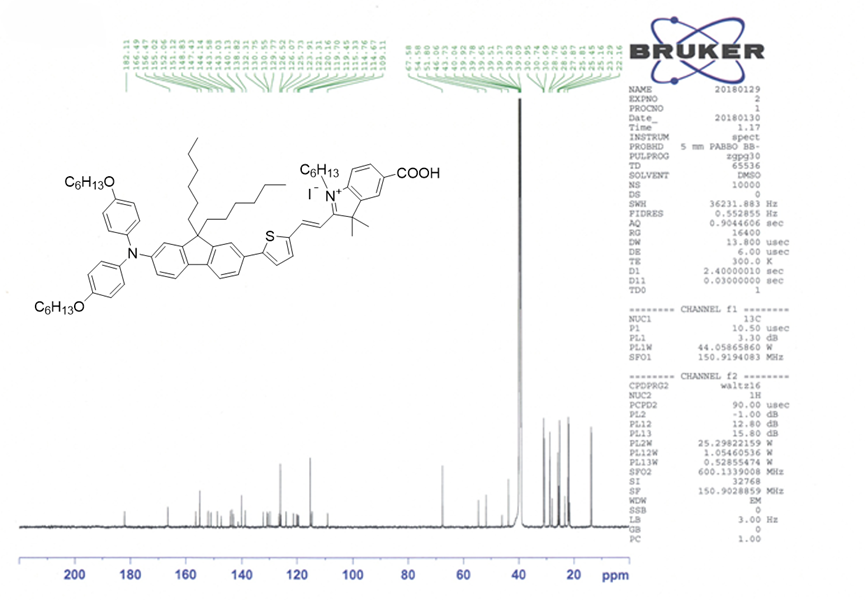


**Fig. S15.** ^1^H (top) & ^13^C NMR (bottom) spectra of **YI-1** in DMSO-d_6_.


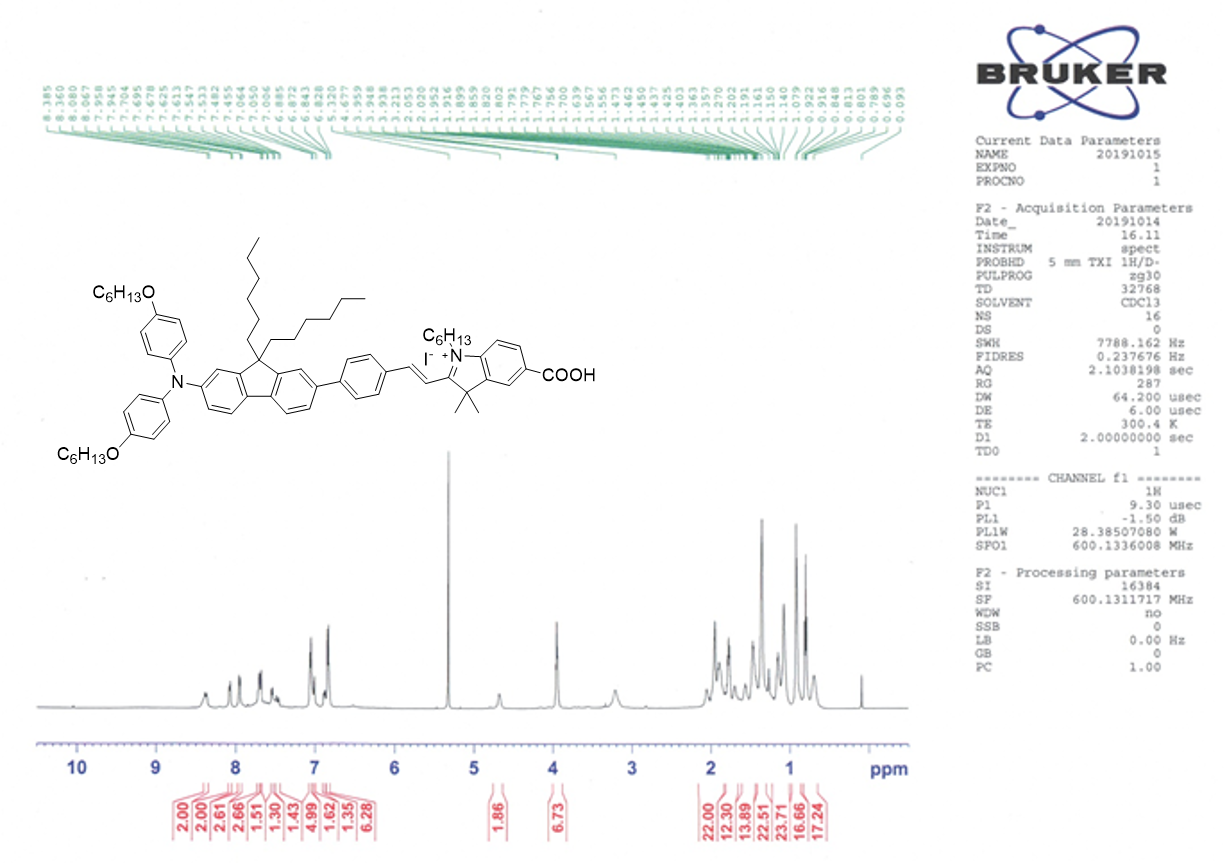


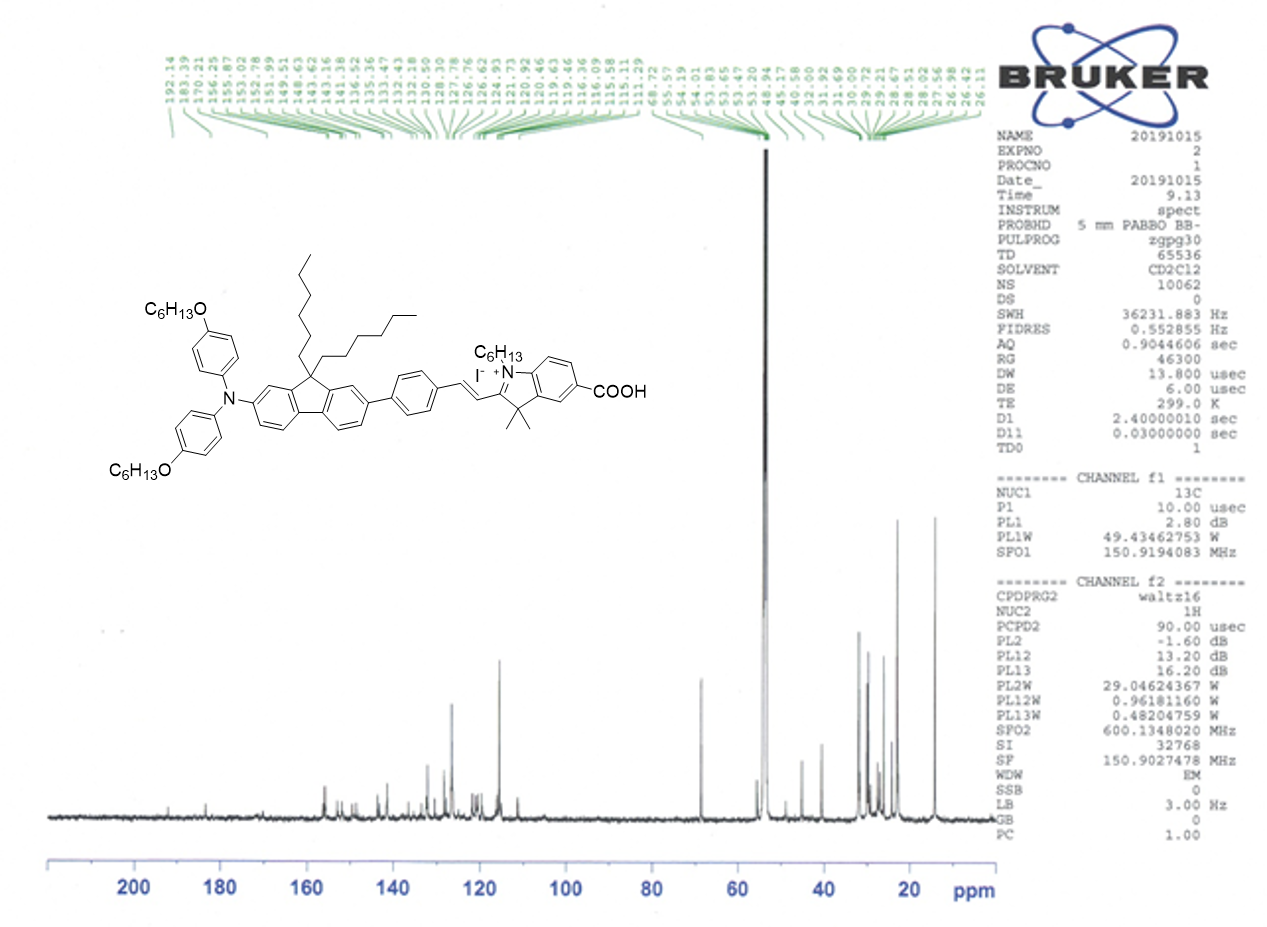


**Fig. S16.** ^1^H (top, in CDCl_3_) & ^13^C NMR (bottom, in CD_2_Cl_2_) spectra of **YI-3**.


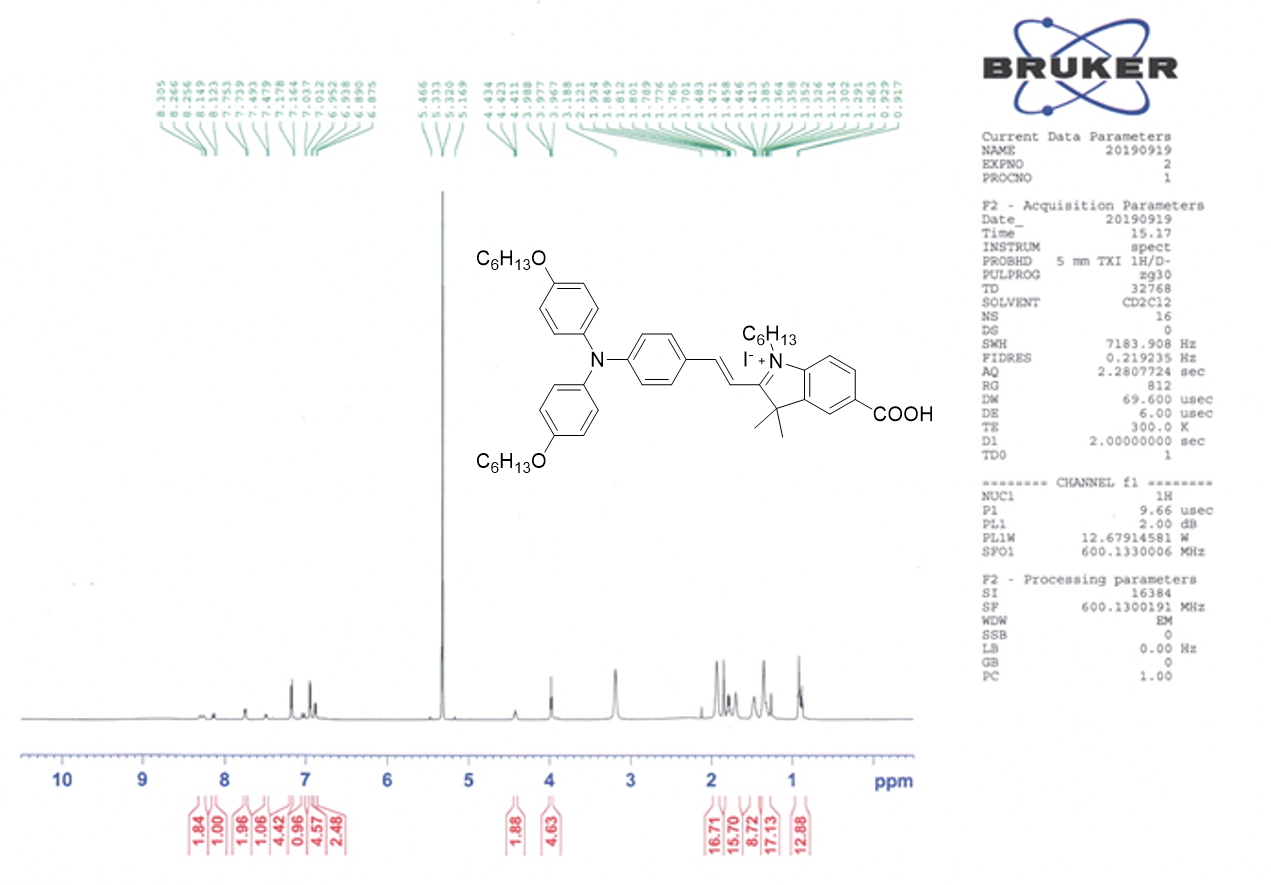


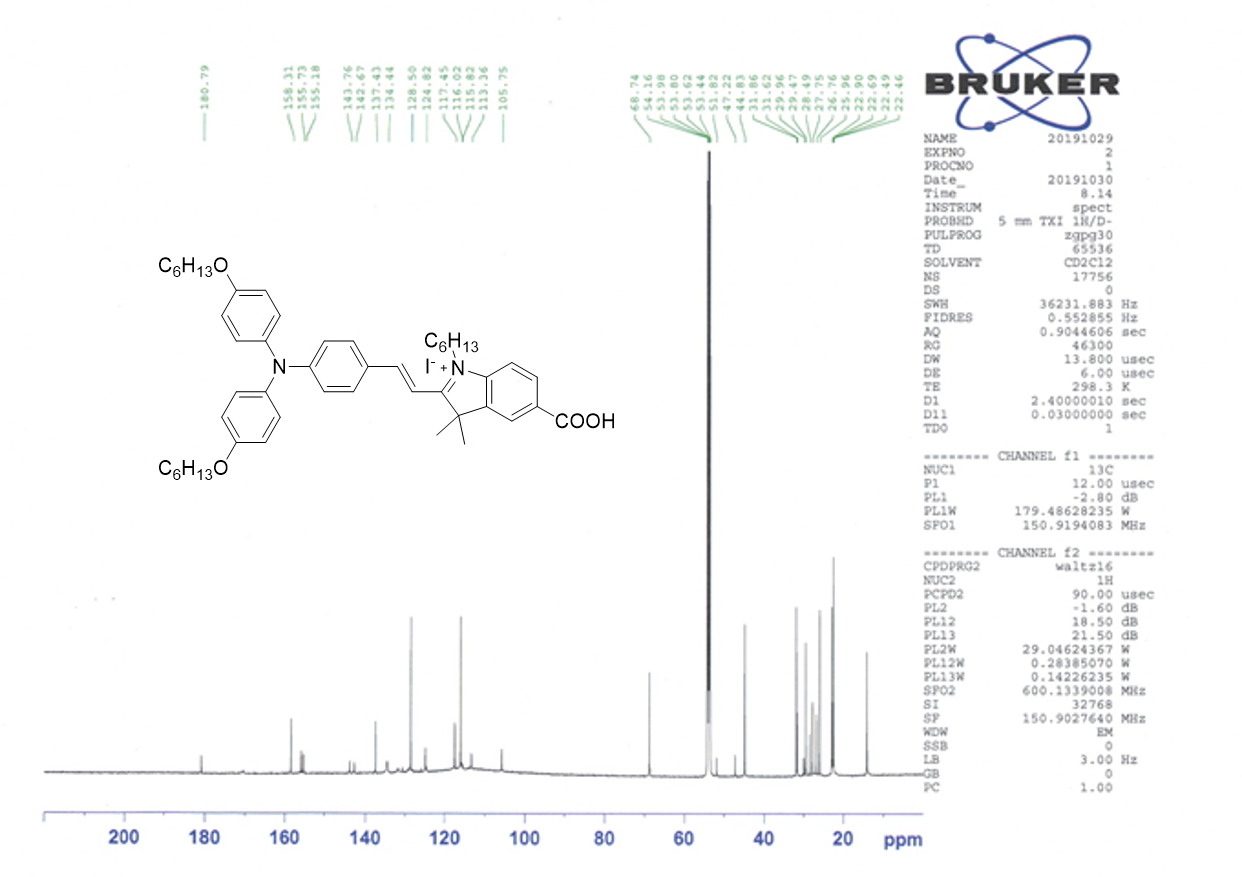


**Fig. S17.** ^1^H (top) & ^13^C NMR (bottom) spectra of **YI-8** in CD_2_Cl_2_.

**Fig. S18**. (a) The absorption rates and (b) loading amounts of different dyes in the PSMA NPs.

**Fig. S19**. The corresponding UV-VIS/fluorescent spectra of (a) ADF3/PSMA@ADF3 (blue fluorescent) and (b) ADF2/PSMA@ADF2 (green fluorescent), respectively.

**Synthesis of DTDPTID**

**Scheme S2.** Synthetic pathway for **DTDPTID**

A mixture of (4-(di-*p*-tolylamino)phenyl)boronic acid (164 mg, 0.516 mmol), tetrakis(triphenylphosphine)palladium(0) (13.6 mg, 0.150 mmol), potassium carbonate (97.3 mg, 0.705 mmol) and **TID-Br** (164 mg, 0.516 mmol) was dissolved in anhydrous toluene (1.2 mL) and nitrogen-bubbled deionized water (0.4 mL). The solution was heated to 110 °C overnight under the protection of argon atmosphere. The solvent was removed with rotary evaporation and the resulting solid was dissolved in dichloromethane and was washed with brine and water. The organic layer was further purified by column chromatography with CH_2_Cl_2_/hexanes (v/v = 2/1) as eluent to afford **DTDPTID** (96.0 mg, 48%) as a purple solid. ^1^H NMR (400 MHz, CDCl_3_) δ 7.72 – 7.63 (m, 4H), 7.28 – 7.16 (m, 20H), 3.68 (d, *J* = 7.5 Hz, 2H), 2.44 (s, 12H), 2.05 – 1.90 (m, 1H), 1.48 – 1.33 (m, 8H), 0.97 (q, *J* = 7.3 Hz, 6H). ^13^C NMR (101 MHz, CDCl_3_) δ 166.68, 157.39, 149.38, 144.57, 133.64, 133.49, 131.99, 130.07, 125.96, 123.48, 118.96, 77.32, 77.00, 76.69, 42.72, 37.93, 30.63, 28.52, 23.99, 23.04, 20.89, 14.03, 10.51. HRMS (m/z, MALDI, [M]^+^) calcd for C_56_H_53_N_5_O_2_S 859.3914 found 859.3948.


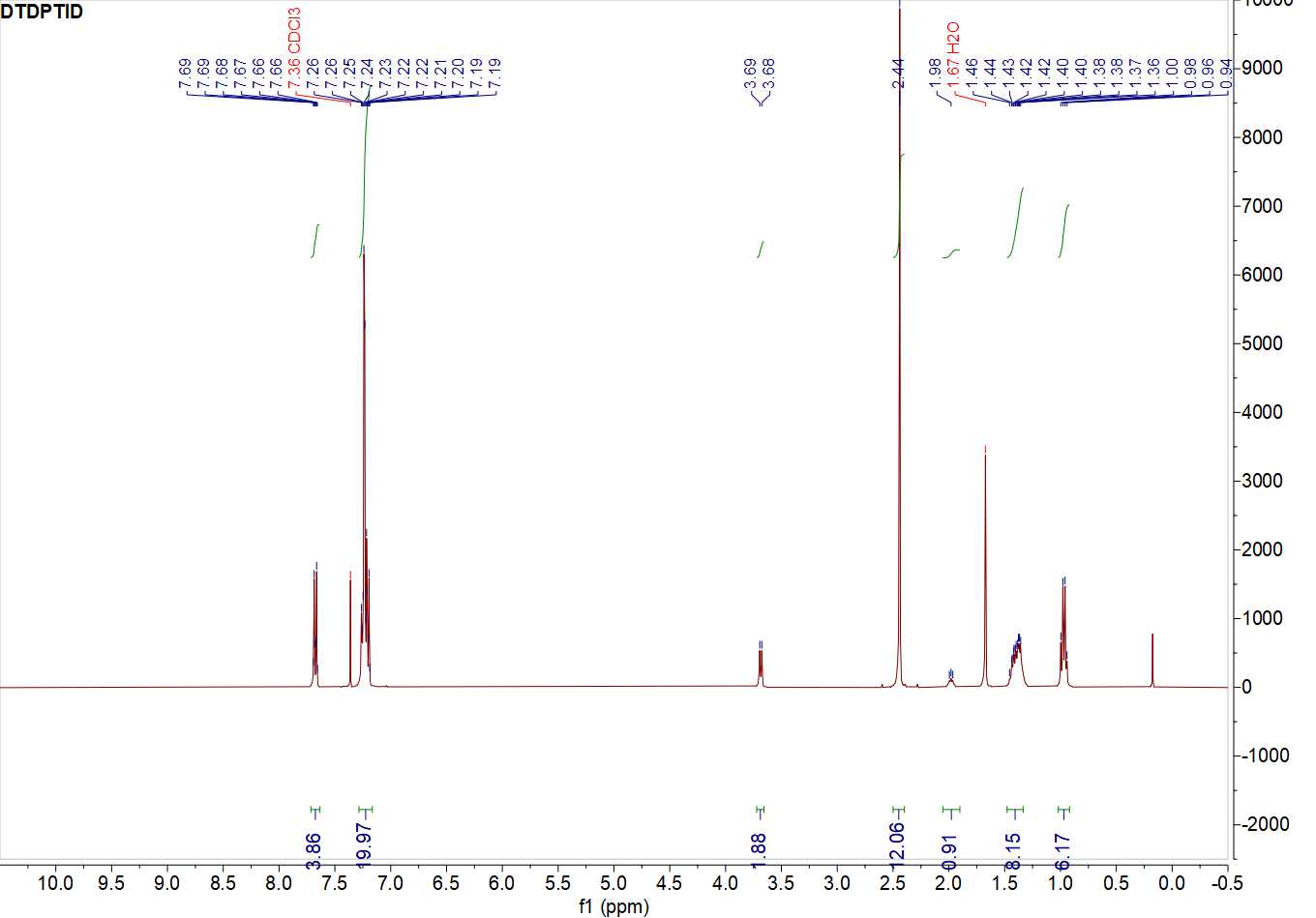


**Fig. S20.** ^1^H spectra of **DTDPTID**.


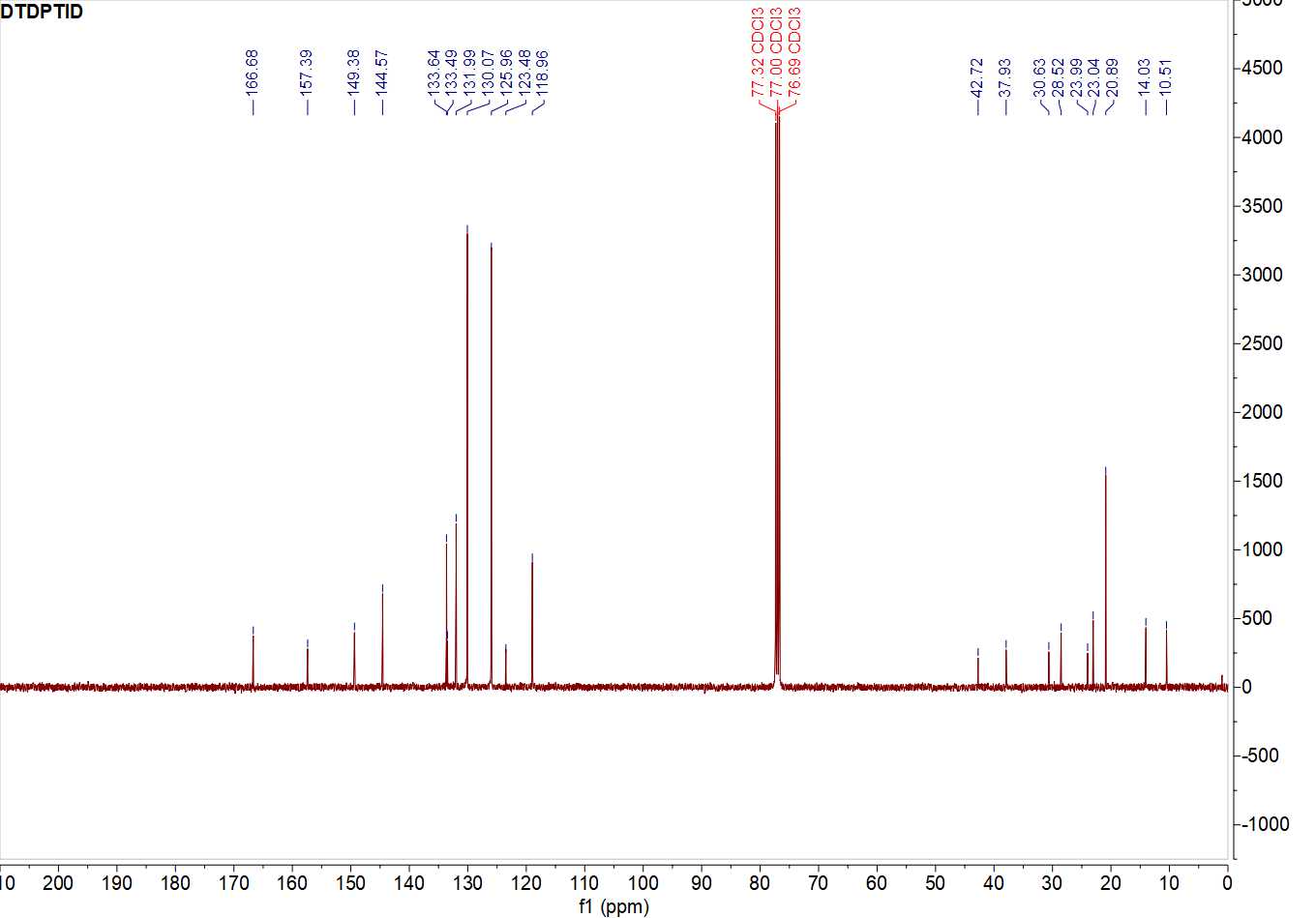


**Fig. S21.** ^13^C spectra of **DTDPTID**.

**Fig. S22**. Multiphoton fluorescnece spectra of **PSMA-DTDPTID NPs.**


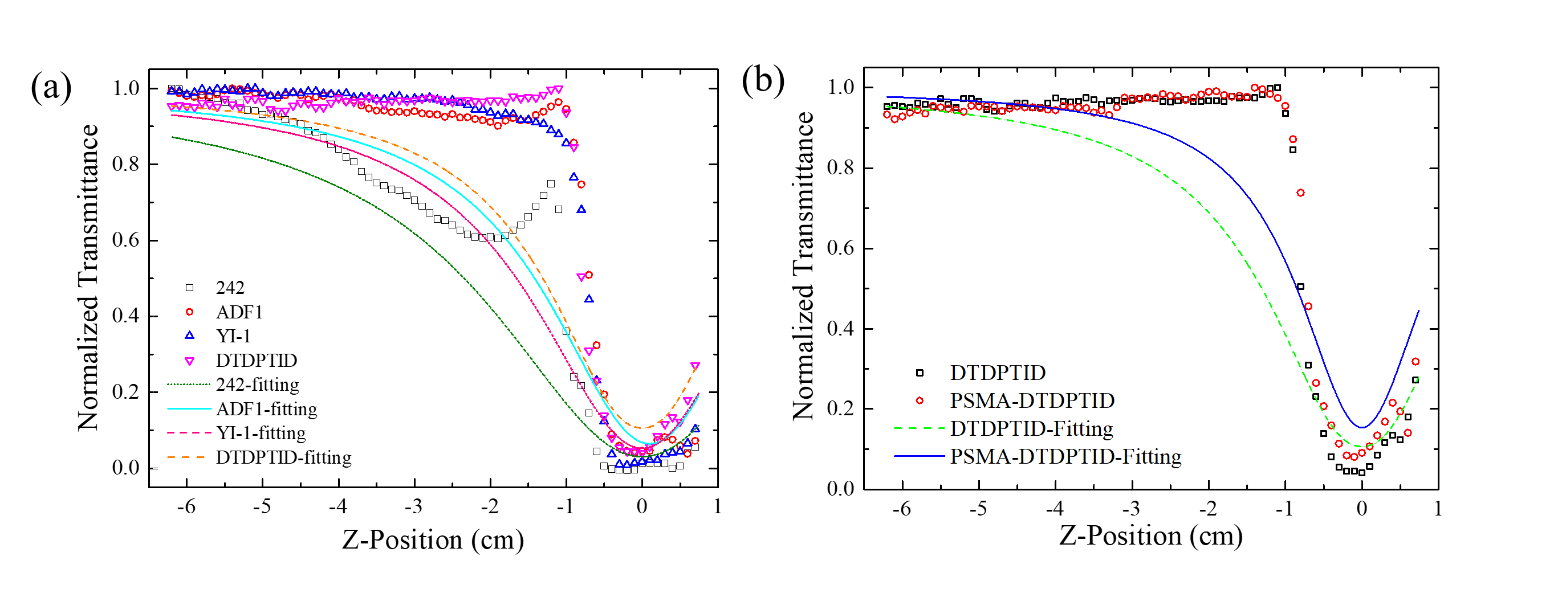


**Fig. S23**. Open aperture Z-scan data and fitting traces of (a) normalized transmittance for 242, ADF1, YI-1, and DTDPTID, and (b) normalized transmittance for DTDPTID and PSMA-DTDPTID, respectively.

 **Fig. S24**. The FTIR spectra of (a) PSMA NPs, (b) pure dyes (242, YI-1 and ADF1) and dye-immobilized PSMA NPs.


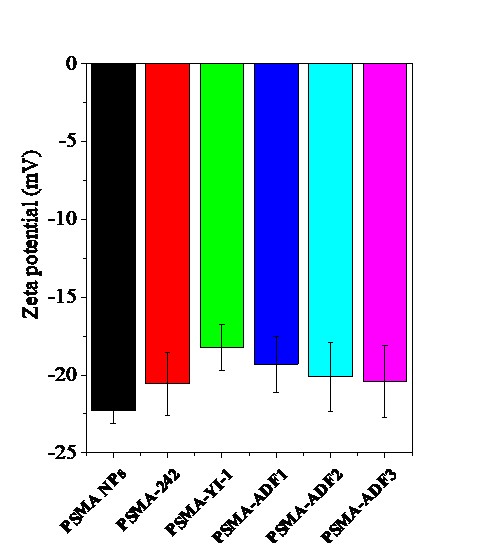


**Fig. S25**. The zeta-potential of PSMA and PSMA-dyes NPs.


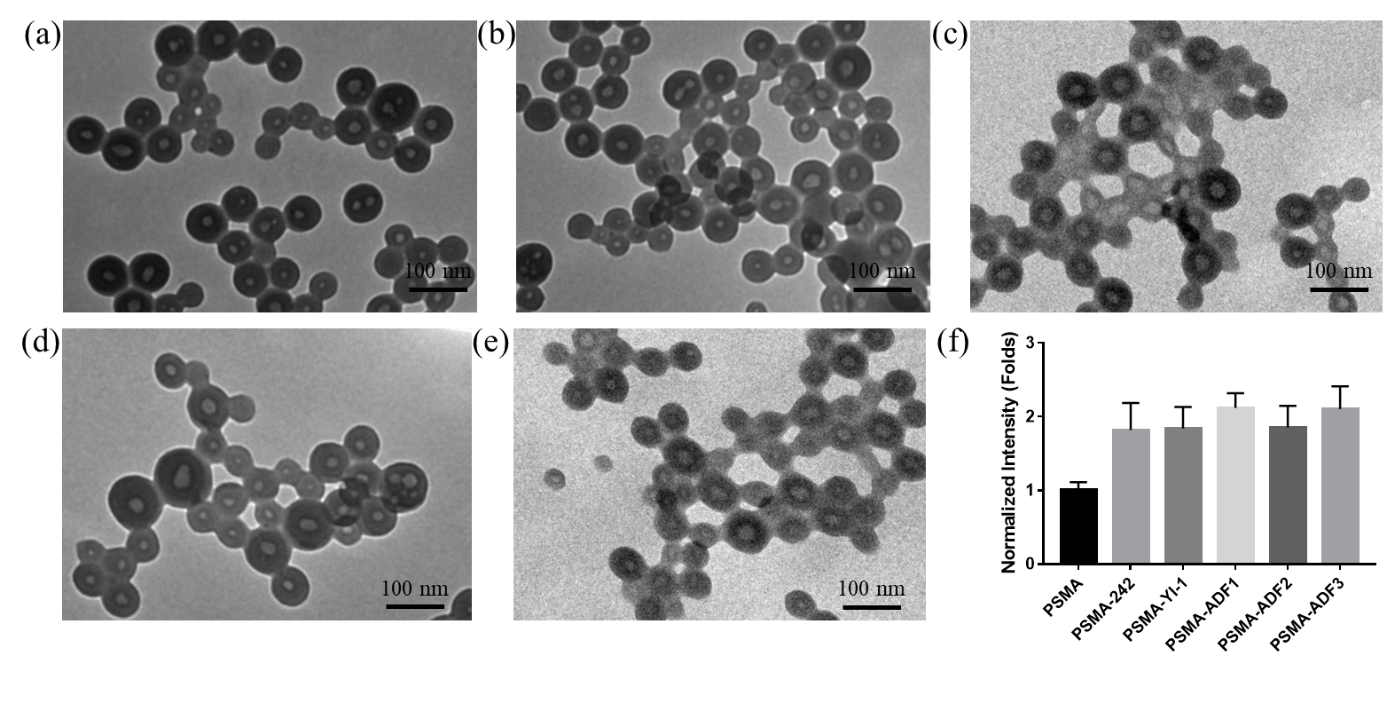


**Fig. S26**. The TEM images of (a) PSMA-242, (b) PSMA-YI-1, (c) PSMA-ADF1, (d) PSMA-ADF2 and (e) PSMA-ADF3, and (f) the quantified contrast intensity (with ImageJ software) of poymer shell matrix (without the selection area in the polymer core center) of the PSMA-dyes compared to the polymer shell matrix of the PSMA NPs alone (Figure 2a in the main text).


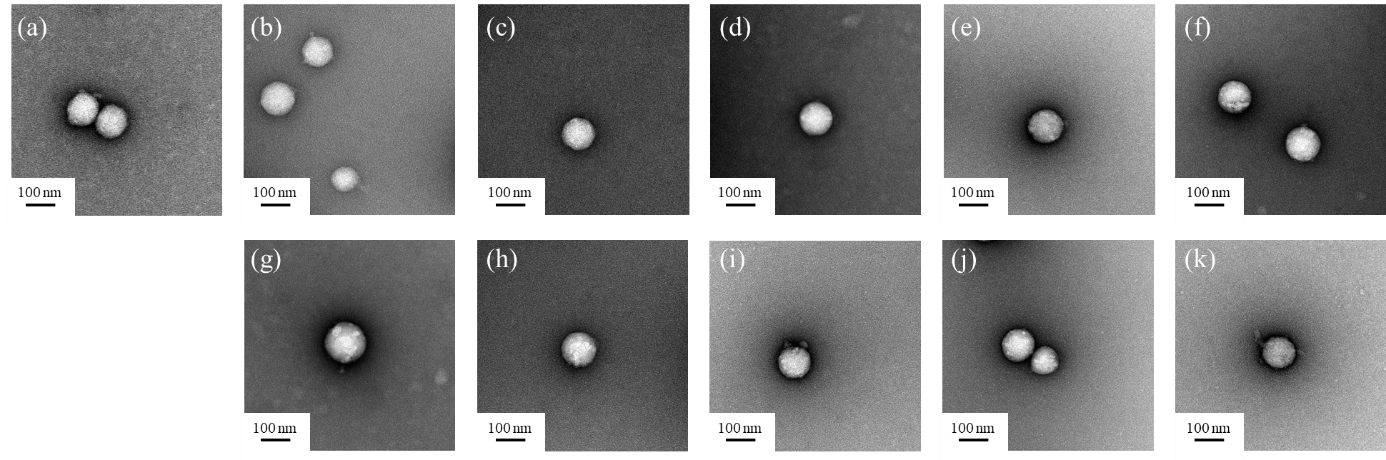


**Fig. S27**. The TEM negative stain images of (a) PSMA, (b) PSMA-FeChl, (c) PSMA-R6G, (d) PSMA-Cy5, (e) PSMA-242, (f) PSMA-YI-1, (g) PSMA-YI-3, (h) PSMA-YI-8, (i) PSMA-ADF1, (j) PSMA-ADF2 and (k) PSMA-ADF3.

**Fig. S28**. (a) The 48h releasing profiles of PSMA-dyes NPs. (b) DLS measurements for the PSMA-dyes size changes in the PBS solution. (c) The cell viability of MB49 cells under treatment of the same amount of released dyes.

**Fig. S29**. The cell images of JC-1 mitochondria membrane potential staining for mitochondria damage evaluation. MB49 cells treated PSMA-242, PSMA-YI-1 and PSMA-ADF1 NPs for 24 h and analyzed for green fluorescence observation (excitation/emission = 488/529 nm).

**Fig. S30**. (a) The confocal microscopy image of MB49 cells treated with PSMA-ADF1 (red) and stained by lysotracker (green). Results showed that the most of the merged fluorescence were yellow and orange colors, suggesting the position of the internalied PSMA-ADF1 and lysosomes were highly co-locolized. Scale bar = 20 μm. (b) The cell imaging of PSMA-ADF1, PSMA-ADF2, and PSMA-ADF3 fluorescence in MB49 cells using bright-field and fluorescent microscopy.

**
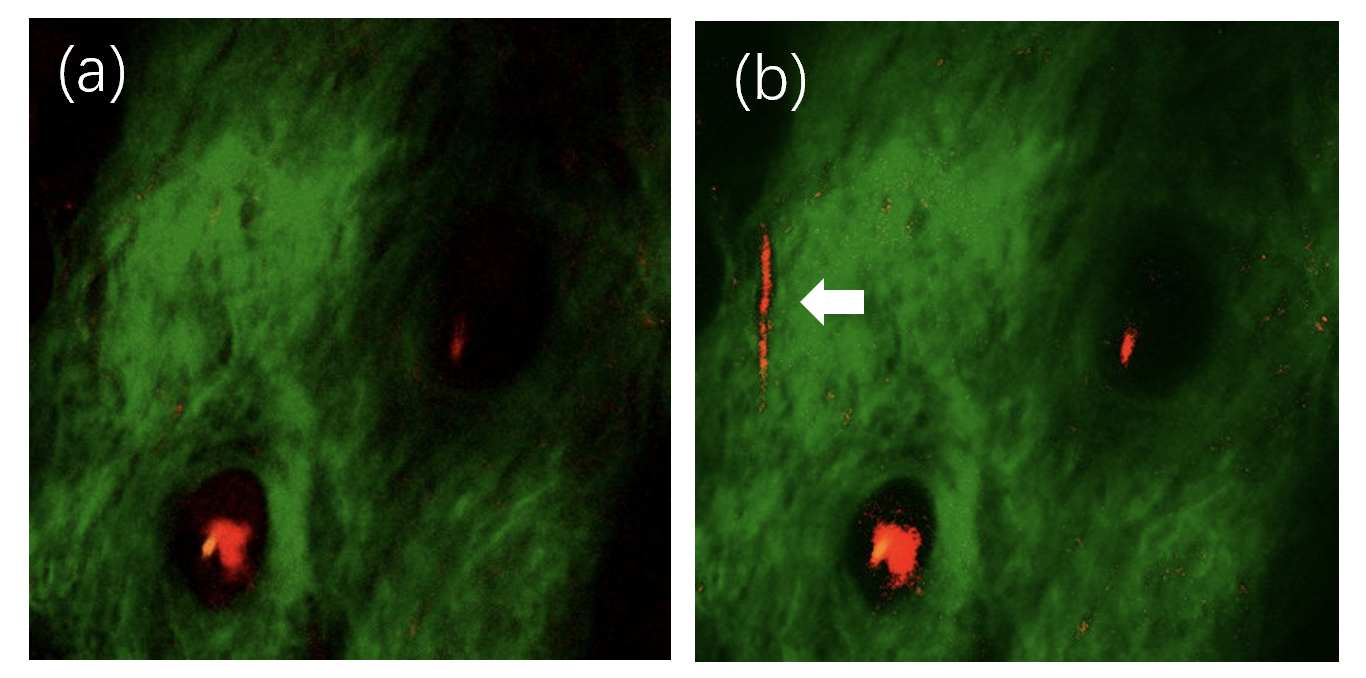
**

**Fig. S31.** *In vivo* two-photon fluorescence (red color) and second harmonic generation (green color) imaging of a mouse ear (a) before and (b) after the tail-vein injection of PSMA-DTDPTID NPs. The white arrow in (b) indicates the region of vessel. The 1080 nm excitation power is 1/3 of that in Figure 4f and 4g. Fields of view: 317 × 317 μm.

**Fig. S32**. The fluorescence changes of PSMA-YI-1 and pure YI-1 dyes in PBS (pH = 4, 7) for 7 days.

**Fig. S33**. The singlet oxygen generation of YI series dyes by RNO/imidazole assay under 660 (solid lines, 75 mW/cm^2^) and 808 (dotted lines, 1W/cm^2^) nm laser irradiation for 10 min.

**Fig. S34**. The thermal curve of PSMA-YI-1 under 808 nm laser irradiation with 0.9 W/cm^2^ power density

**Fig. S35**. The singlet oxygen generation of PSMA, PSMA-242 and PSMA-ADF1 by RNO/imidazole assay under 660 (solid lines, 75 mW/cm^2^) and 808 (dotted lines, 0.9 W/cm^2^) nm laser irradiation for 10 min.

**Fig. S36**. The H&E stain of major organ sections (heart, liver, spleen, lung and kidney) from PBS or PSMA-YI-1 NPs treated mice. Scale bar: 100 μm.

**Table S1**. Fitting parameters, saturable intensities (*I*_S_) and TPA coefficients (*β*), of open aperture Z-scan measurement for 242, ADF1, YI-1, DTDPTID and PSMA-DTDPTID, respectively.

|  | *I*_S_ (GW/cm^2^) | *β* (cm/W) |
| --- | --- | --- |
| 242 | 0.10 | 7.13×10^-8^ |
| ADF1 | 0.23 | 5.11×10^-8^ |
| YI-1 | 0.20 | 5.38×10^-9^ |
| DTDPTID | 0.30 | **1.19×10^-6^** |
| PSMA-DTDPTID | 0.64 | 2.93×10^-8^ |

**Table S2**. The size and zeta potential of different PSMA-dyes and their core pore diameter.

|  | PSMA | Cu@PSMA | PSMA-242 | PSMA-YI-1 | PSMA-ADF1 |
| --- | --- | --- | --- | --- | --- |
| Diameter (nm) | 73.4±11.9 | 74.8±8.3 | 81.2±14.4 | 80.8±14.6 | 74.9±12.5 |
| Pore diameter (nm) | 17.1±6.6 | 19.9±5.9 | 24.4±5.1 | 23.7±6.2 | 29.4±5.7 |
| Zeta potential (mV) | -22.3±0.8 |  | -20.6±2.0 | -18.2±1.5 | -19.31.8 |
